# Polysaccharide Nanoparticles for Efficient siRNA Targeting in Cancer Cells by Supramolecular p*K*_a_ Shift

**DOI:** 10.1038/srep28848

**Published:** 2016-07-01

**Authors:** Ying-Ming Zhang, Yang Yang, Yu-Hui Zhang, Yu Liu

**Affiliations:** 1 Department of Chemistry, State Key Laboratory of Elemento-Organic Chemistry, Nankai University, Tianjin 300071, P. R. China; 2 Collaborative Innovation Center of Chemical Science and Engineering (Tianjin), Tianjin 300071, P. R. China

## Abstract

Biomacromolecular p*K*_a_ shifting is considered as one of the most ubiquitous processes in biochemical events, e.g., the enzyme-catalyzed reaction and protein conformational stabilization. In this paper, we report on the construction of biocompatible polysaccharide nanoparticle with targeting ability and lower toxicity by supramolecular p*K*_a_ shift strategy. This was realized through a ternary assembly constructed by the dual host‒guest interactions of an adamantane-bis(diamine) conjugate (ADA) with cucurbit[6]uril (CB[6]) and a polysaccharide. The potential application of such biocompatible nanostructure was further implemented by the selective transportation of small interfering RNA (siRNA) in a controlled manner. It is demonstrated that the strong encapsulation of the ADA’s diammonium tail by CB[6] not only reduced the cytotoxicity of the nano-scaled vehicle but also dramatically enhanced cation density through an obvious positive macrocycle-induced p*K*_a_ shift, which eventually facilitated the subsequent siRNA binding. With a targeted polysaccharide shell containing a cyclodextrin‒hyaluronic acid conjugate, macrocycle-incorporated siRNA polyplexes were specifically delivered into malignant human prostate PC-3 cells. The supramolecular polysaccharide nanoparticles, the formation of which was enabled and promoted by the complexation-assisted p*K*_a_ shift, may be used as a versatile tool for controlled capture and release of biofunctional substrates.

The states of protonation in solution, which can be quantitatively measured by the acidity constant of p*K*_a_, have critical relevance to our biomedical and physiological events^1^,^2^,^3^,^4^. The specific function of a given chemical group as a nucleophiles, electrophiles, or general acid−base catalysts in myriad of biological reactions can largely lies in its state of protonation. Significantly, the precise control of prototropic equilibrium can also profoundly affect the electrostatic stabilization of native biomacromolecules by facilitating the favorable opposite-charge attraction and avoiding the unfavorable Coulombic repulsion. The primary electrostatic attraction originating from p*K*_a_ shifting is vital to many biological processes, by which some secondary interactions, such as hydrophobic and hydrogen bonding interactions, can be further stimulated to propagate the formation of well-organized superstructures and to eventually achieve diverse biological functions^5^,^6^,^7^,^8^. Thus, the elaborate optimization and implementation of electrostatic force through p*K*_a_ regulation is useful in molecular-level understanding of the multiple noncovalent contributions in biology-related systems.

Recent studies of supramolecular chemistry have significantly improved our understanding of noncovalent interactions, which is a prerequisite for mimicking various biological processes^9^,^10^,^11^,^12^. In this regard, the use of synthetic macrocycles has greatly expanded the roles of chemical synthesis and the range of methodologies employed in modern nanomedicine^13^,^14^,^15^,^16^,^17^. In particular, macrocycle-based supramolecular approach may provide a convenient and powerful tool for the improvement of biocompatiblility and reducing side effects, mainly due to its immense advantages to regulate the physicochemical properties of individual subunits or components by integrating the multiple noncovalent interactions^18^,^19^,^20^,^21^,^22^. It is also noteworthy that macrocyclic encapsulation has been proven to alter chemical reactivity and modify the protonation–deprotonation equilibria of included small molecules through a complexation-induced p*K*_a_ shift method^23^,^24^. Therefore, one can believe that the rational combination of macrocyclic receptors with efficient p*K*_a_ regulation will be developed into a promising approach to bind ionized bioactive substrates in a controlled manner.

Moreover, there is a general consensus that the binding process with nucleic acids always occurs via electrostatic interaction, which is ubiquitously practiced as the reliable noncovalent driving force owing to the inherently anionic property of naked nucleic acids^25^,^26^. This principle inspired us to hypothesize that a controlled nucleic acid binding may be realized by implementing the complexation-induced p*K*_a_ shift strategy to adjust the acid−base equilibria in appropriate guests. In this work, the application of supramolecular p*K*_a_ shift was implemented by the controlled binding of nucleic acids. Through a large and positive cucurbituril-induced supramolecular p*K*_a_ shift, we found that small interfering RNA (siRNA) can be readily assembled into a macrocycle-based nanosystem for targeted delivery into cancer cells. The association of cucurbituril could greatly facilitate the electrostatic attraction and the protonation of polyamine groups in a triad guest at a neutral pH and significantly increases the cationic density for efficient siRNA packaging. Meanwhile, the biodegradable cyclodextrin-tethered hyaluronic acid shell, which is specifically recognized by hyaluronic acid receptors that are overexpressed on the surface of cancer cells, enhances the biocompatibility of the overall delivery system while ensuring targeting capability^27^,^28^. Besides the desirable targeting capability, the siRNA-bound polyplexes, which are derived from the selective bimodal molecular recognition of the adamantane-bis(diamine) conjugate (ADA) with *β*-cyclodextrin (*β*-CD) and cucurbit[6]uril (CB[6]), exhibit higher transfection efficiency than that observed for the individual ammonium guest and conventional transfection reagents. It can be anticipated that the effective regulation of the p*K*_a_ value of the amino group in ADA by host–guest complexation and the selective delivery of nucleic acids in target cells may enable practical applications of macrocycle-based non-viral vectors in gene therapy. Although some relevant studies on protonation-directed gene silence and hyaluronic acid-based delivery systems have been separately reported^29^,^30^,^31^, we herein report a quite simple and practical method to precisely adjust the cation density of polysaccharide nanoparticles by supramolecular p*K*_a_ shift. To the best of our knowledge, this is the first example of implementing macrocycle-induced p*K*_a_ shift concept in the selective transportation of nucleic acids.

## Results and Discussion

### Synthesis of CB[6]‒ADA‒HACD assembly

**Figure 1:**
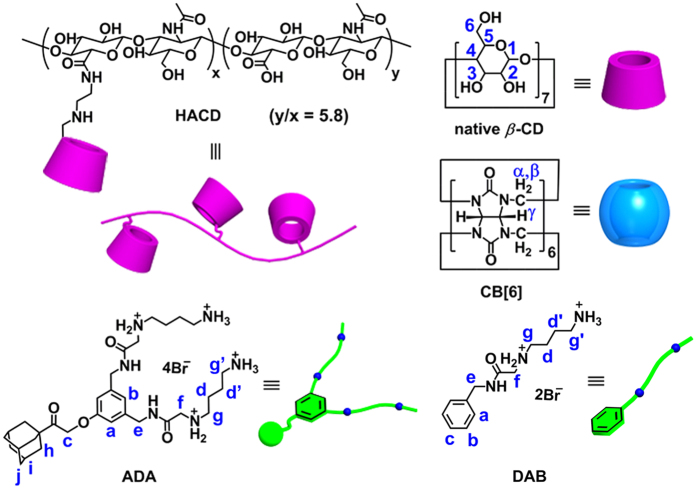
Molecular structures and proton designations of ADA, DAB, HACD and CB[6]. HACD carries *β*-CD at every 6.8 repeating units. ADA carries one adamantyl head and two polyammonium tails and is simultaneously bound to *β*-CD and CB[6].

*β*-CD-modified hyaluronic acid (HACD) carries 17 *β*-CD units on average in a HA chain and functions as the polysaccharide shell. HACD was prepared by the amidation of hyaluronic acid with mono-6-deoxy-6-(2-aminoethylamino)-*β*-CD, according to procedures reported previously^32^. The degree of substitution employed was suitable for balancing the specific recognition by HA receptor-overexpressing cancer cells and the multivalent binding of hydrophobic substrates. The triad guest molecule (ADA, **1**) carries one adamantyl moiety and two protonated 1,4-diamine chains. ADA was prepared in 4 steps from adamantyl bromomethyl ketone (**2**) and the pentafluorophenyl ester of Cbz-protected 4-aminobutylaminoacetic acid (**6**) (see Supplementary Figs S1−S18). As illustrated in Fig. 1, ADA carries two types of independent guest moieties. One is the bulky adamantyl group to be included in the *β*-CD cavity; the other is the 1,4-diammonio moieties that are to be bound to CB[6]. The pendant polycationic sidechains of ADA are also expected to electrostatically interact with the anionic phosphate backbones of siRNA^33^,^34^,^35^. A ternary CB[6]‒ADA‒HACD complex that benefited from the bimodal binding of ADA with two different types of macrocycles and was able to target cancer cells was readily and selectively obtained.

### Molecular Binding Behaviors of ADA with *β*-CD and CB[6]

Before examining complexation with nucleic acid, the affinities between ADA and HACD and CB[6] were evaluated by NMR spectroscopy. Native *β*-CD was used as a reference for HACD. The binding stoichiometry was determined as 1:1 for *β*-CD‒ADA complexation via a Job analysis of the peak shifts of the adamantyl H_h_ proton originally located at *δ* 1.95 ppm (see Supplementary Fig. S19). Using 1:1 stoichiometry, the association constant (*K*_a_) was determined to be 6.09 × 10^4^ M^−1^; the chemical shift changes of the adamantane proton of ADA were analyzed upon titration with native *β*-CD (see Supplementary Fig. S20). As this *K*_a_ value obtained is comparable to or even larger than those reported for adamantanols and adamantanecarboxylates, which typically range from 10^3^ to 10^5^ M^−1^, the polyammonium sidechains in ADA do not appear to significantly affect the intermolecular complexation of ADA by *β*-CD^36^,^37^. Meanwhile, the butane-1,4-diamine derivative bearing single ammonium arm (DAB) was employed as the reference molecule to evaluate the complexation behaviors between CB[6] and the ammonium sidechain (see Supplementary Figs S21–S26). Isothermal titration calorimetry (ITC) measurements showed that this binding process was governed in a thermodynamically favorable way with negative enthalpy and positive entropy changes, accompanied by the high binding affinity up to 10^6^ M^−1^ (see Supplementary Fig. S27). These molecular binding parameters reveal that the bifunctional ADA molecule could be concurrently associated with *β*-CD and CB[6] to form a stable inclusion complex in water, and more importantly, this extremely strong binding would facilitate the eventual formation of siRNA polyplexes, as described below.

**Figure 2:**
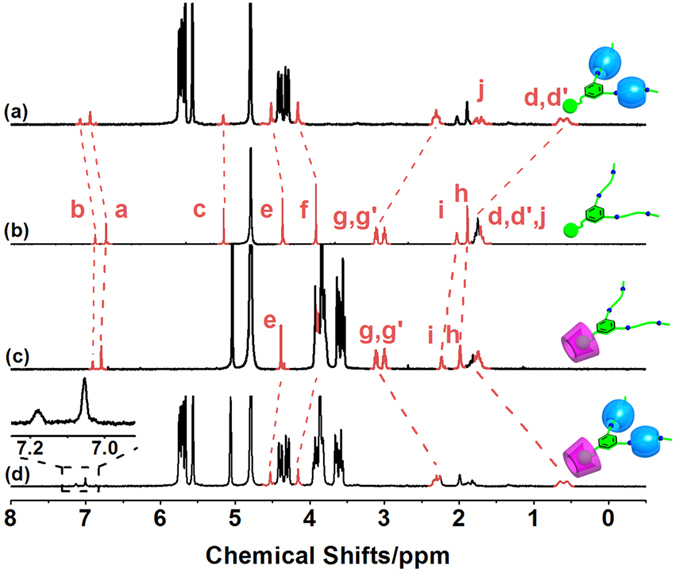
^1^H NMR titration of DAB with *β*-CD and CB[6] in the molecular self-assembling process. ^1^H NMR spectra at 400 MHz of (**a**) ADA‒CB[6] complex, (**b**) ADA, (**c**) ADA‒*β*-CD complex, and (**d**) *β*-CD‒ADA‒CB[6] complex in D_2_O at 25 °C. Inset in (d): Magnified area of phenyl protons ([ADA] = 1.0 mM, [CB[6]] = 2.0 mM, and [*β*-CD] = 3.0 mM).

Figure 2a‒d illustrate the individual and simultaneous binding behaviors of ADA with *β*-CD and/or CB[6]. The characteristic peaks of the ADA’s aliphatic tails shifted to the upper field upon complexation with CB[6]; the H_d,d_, and H_g,g_, protons displayed pronounced complexation-induced upfield shifts of 1.13 and 0.78 ppm, respectively. Conversely, the adjacent H_a,b,e,f_ protons showed downfield shifts. The adamantyl H_h,i_ protons were nearly unaffected in the presence of CB[6]. These contrasting NMR spectral shifts demonstrate that the pendant diammonium sidechains are tightly bound to CB[6] through an ion-dipole interaction; the aromatic and adamantane residues remain unaffected outside the cavity of CB[6] (Fig. 2a,b)^38^.

In the case of the *β*-CD–ADA complex, the H_d,d_, and H_g,g_, protons in the sidechain were completely unaffected. The aromatic H_a_ and H_b_ protons of ADA shifted downfield only slightly (0.06 and 0.04 ppm, respectively). The adamantyl H_h_ proton was subject to a larger downfield shift of 0.11 ppm due to the hydrophobic interactions of the adamantine moiety in the *β*-CD cavity (Fig. 2b,c). When both of the macrocycles (*β*-CD and CB[6]) were added to an ADA solution, the overall chemical shift changes of the ADA protons (Fig. 2d) were nearly equivalent to the sum of the shift changes caused independently by CB[6] (Fig. 2a) and *β*-CD (Fig. 2c); the observed additivity of the chemical shift changes suggests the non-interfering complexation of both macrocycles with ADA.

Nuclear Overhauser enhancement spectral (NOESY) examinations revealed NOE cross-peaks between the H_h,i_ protons of adamantane and the H_3,5_ protons of the CD interior (peaks A and B in Supplementary Fig. S28). Furthermore, the NOE cross-peaks between the CB[6]’s H_*α*, γ_ protons and the *β*-CD’s H_2,3_ protons (peaks C and D in Supplementary Fig. S28) provide strong evidence for the coexistence of two macrocycles in close proximity in the ternary complex. These ^1^H NMR titration results unequivocally corroborate that a ternary *β*-CD‒ADA‒CB[6] complex is exclusively formed through simultaneous specific binding of the adamantyl head of ADA by *β*-CD and the diammonium tails by CB[6].

### Supramolecular p*K*_a_ shift of ADA‒CB[6] complex

**Figure 3:**
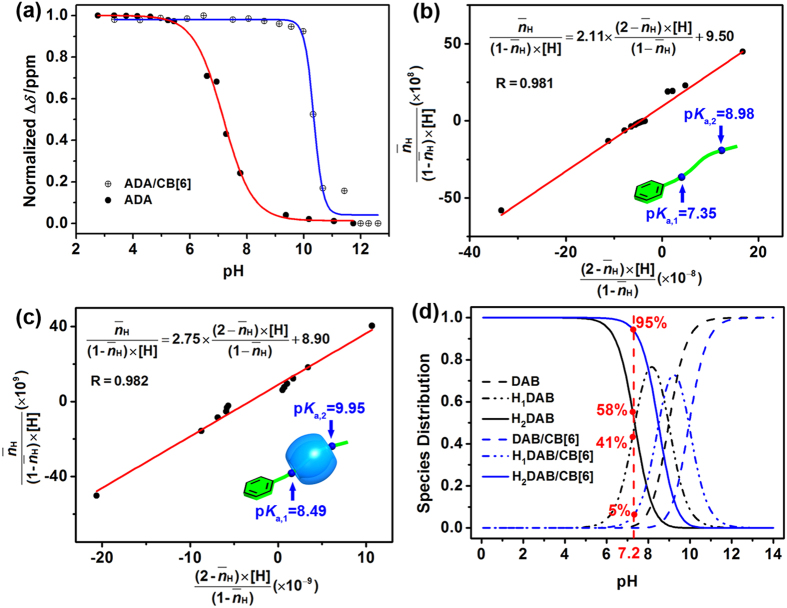
Supramolecular p*K*_a_ shift of DAB‒CB[6] complex. (**a**) ^1^H NMR titration curves for free ADA and ADA‒CB[6] complex by monitoring H_f_ proton (see Fig. 1) upon variations in pH in aqueous solutions at 25 °C, which revealed a different degree of protonation induced by the complexation of ADA with CB[6] ([ADA] = 0.5 mM and [CB[6]] = 1.0 mM); for the original data, see Supplementary Fig. S29; potentiometric fitting curve and p*K*_a_ values calculation of (**b**) DAB and (**c**) DAB‒CB[6] system ([DAB] = 0.5 mM and [CB[6]] = 1.0 mM); (**d**) pH-dependent species distribution plot for the DAB‒CB[6] system. The distributions were obtained using the calculated p*K*_a,1_ and p*K*_a,2_ values. The H_1_DAB and H_2_DAB indicate the singly and doubly protonated species of DAB, respectively.

As synthetic vectors for nucleic acid binding mediated by ammonium ions are very sensitive to the pH in solution, it is essential to take into account the pH effect. The optimal charge density (or number of charges) in electrostatically driven complexation with biomacromolecules must also be determined^39^,^40^,^41^,^42^. In our case, the H_f_ proton in ADA and the ADA–CB[6] complex was very sensitive to the pH in solution; therefore, this proton was monitored during the pH titration experiments. As discerned from the plot of chemical shifts versus pH, it is found that the titration curve was significantly shifted to higher pH region in ADA–CB[6] complex, suggesting that the degree of protonation in ADA would be greatly changed in the presence of CB[6] (Fig. 3a and Supplementary S29). Moreover, it can be seen that the corresponding chemical shift changes of DAB took on similar characteristics in the protons of ADA upon association with CB[6]; that is, the protons of diamine tail (H_d,d’_ and H_g,g_) underwent a pronounced upfield shift (−1.08 and −0.69 ppm, respectively), whereas the ones of aromatic head (H_a−c_) were almost unaffected (see Supplementary Fig. S30). These ^1^H NMR chemical shift changes demonstrate that the ammonium sidechains in ADA and DAB could be exclusively encapsulated by CB[6]. Therefore, DAB was used as the reference compound to quantitatively investigate the complexation-induced p*K*_a_ changes in the potentiometric titrations (Fig. 3b,c). The results clearly showed that there was an obvious p*K*_a_ shift upon complexation with CB[6] (Δp*K*_a,1_ = 1.1 and Δp*K*_a,2_ = 1.0). As shown in Fig. 3d, the species distribution of doubly protonated DAB in the absence and presence of CB[6] was accordingly calculated as 58% and 95%, respectively, in an aqueous solution at pH 7.20 and meanwhile, the content of singly protonated species sharply decreased from 41% to 5%. These results demonstrate that the monocationic DAB could be largely converted into the dicationic one with assistance of CB[6], thus resulting in a high density of positive charges in neutral solution.

Furthermore, complexation-enhanced protonation was confirmed by comparing the ESI mass spectra of ADA in the presence and absence of CB[6]. ADA exhibited a doubly charged parent peak at *m/z* 293 in the absence of CB[6]; however, a quadruple-charged parent peak for the ADA–CB[6] complex was found at *m/z* 645 in the presence of CB[6] (see Supplementary Figs S18 and S31). These results jointly demonstrate that the diamine sidechains of ADA are fully protonated once complexed with CB[6], even in neutral solutions^43^. Crucially, this CB[6]-induced p*K*_a_ shift significantly increases the positive charge density of the resulting assembly under the physiological conditions. This finding was validated by the siRNA binding assay, as described below.

### Characterization of siRNA-bound polyplexes

**Figure 4:**
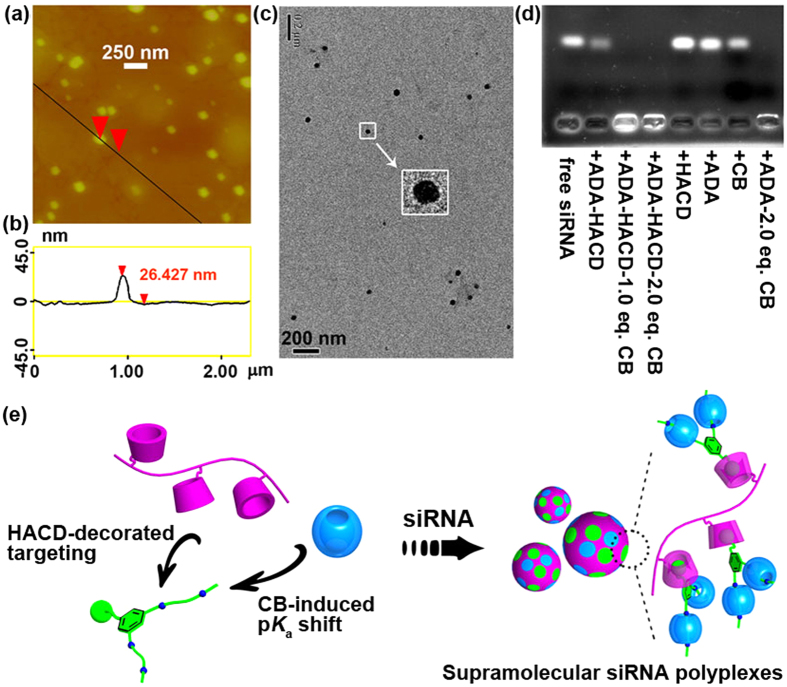
Morphological characterization and electrophoretic mobility of ternary assembly mixed with siRNA. (**a**) AFM image, (**b**) height profile, (**c**) TEM image of siRNA condensed by CB[6]‒ADA‒HACD triads (Inset: magnification); (**d**) agarose gel electrophoresis assay of ADA, CB[6], and their complexes with negative control siRNA; the ADA:CB[6] ratio in (**d**) was fixed at 1:2; and (**e**) schematic illustrations of the assembling process of siRNA with CB[6]‒ADA‒HACD triads.

The enhanced cation density of ADA by the CB[6]-induced p*K*_a_ shift enabled us to prepare siRNA-bound polyplexes by simply mixing solutions of siRNA and the CB[6]‒ADA‒HACD complex. The morphological and structural features of the siRNA-bound polyplexes prepared in this manner were examined by atomic force microscopy (AFM) and transmission electron microscopy (TEM). The original CB[6]‒ADA‒HACD complex appeared as a loose nanoparticulate with a high aspect ratio. Once packed with siRNA, the polyplex become more compact in size; this is due to the neutralization of charges between polyanionic siRNA and the polycationic ternary complex (see Supplementary Figs S32 and S33). As exemplified in Fig. 4a,b, the average height of the polyplex measured by AFM was 25 ± 5 nm. The siRNA aggregates formed upon complexation with the CB[6]‒ADA‒HACD complex were considered to be this size. TEM images revealed a number of solid nanoparticles with an average diameter of 39 ± 4 nm. These findings were in agreement with the average height measured by AFM (Fig. 4c). The spherical morphology and the nanoparticle size observed confirm that the CB[6]‒ADA‒HACD assembly was able to properly condense siRNA. Such condensation facilitates HA receptor-mediated internalization in cancer cells.

Subsequently, siRNA binding behavior was examined by analyzing electrophoretic mobility at different N/P ratios (i.e., the charge ratio of cationic ammonium in ADA to anionic phosphate in siRNA). As shown in Supplementary Fig. S34a, siRNA was completely condensed by the ADA‒CB[6] complex at N/P ≥ 20. Hence, the N/P ratio was fixed at 20 in the following cellular experiments. Additional control experiments were performed to further elucidate the roles of CB[6] in the siRNA binding process. Among the supramolecular hosts and complexes examined, ADA or CB[6] alone did not cause any appreciable condensation (see Supplementary Fig. S34b,c). These findings provided convincing evidence for CB-promoted siRNA condensation and were consistent with the sufficient protonation of ADA after binding with CB[6] under neutral conditions. Interestingly, the condensation effect of the ADA‒CB[6] complex does not appear to be seriously affected by HACD (see lanes 2‒5 in Fig. 4d). This is likely due to the steric hindrance of CB[6] encapsulation at the diammonium sites, which keeps the carboxylate anions of HACD away from the positively charged diammonium sidechains of ADA. In contrast, no free siRNA was observed when mixed with ADA and a sufficient amount of CB[6]. This result confirms that the siRNA-condensing capability of ADA is significantly improved in the presence of CB[6].

### Cytotoxicity of the CB[6]‒ADA‒HACD complex

It is known that high-density of positive charges in transfection reagents used for DNA and RNA delivery can seriously damage cytomembranes and organelles and may lead to serious cytotoxicity toward target cells^44^,^45^,^46^,^47^,^48^,^49^,^50^. First, MTT assay was carried out to evaluate the cytotoxicities of the CB[6]‒ADA‒HACD assembly and Lipofectamine 2000. It was found that the ternary assembly exhibited very high cell viability (>85%) in PC-3 cells at concentrations up to 340 *μ*M. In comparison, Lipofectamine 2000 showed significant cytotoxicity (cell viability < 50%) under the same experimental conditions (see Supplementary Fig. S35). These data demonstrate that the obtained ternary assembly has a much lower cytotoxicity compared with the conventional transfection reagent Lipofectamine 2000.

**Figure 5:**
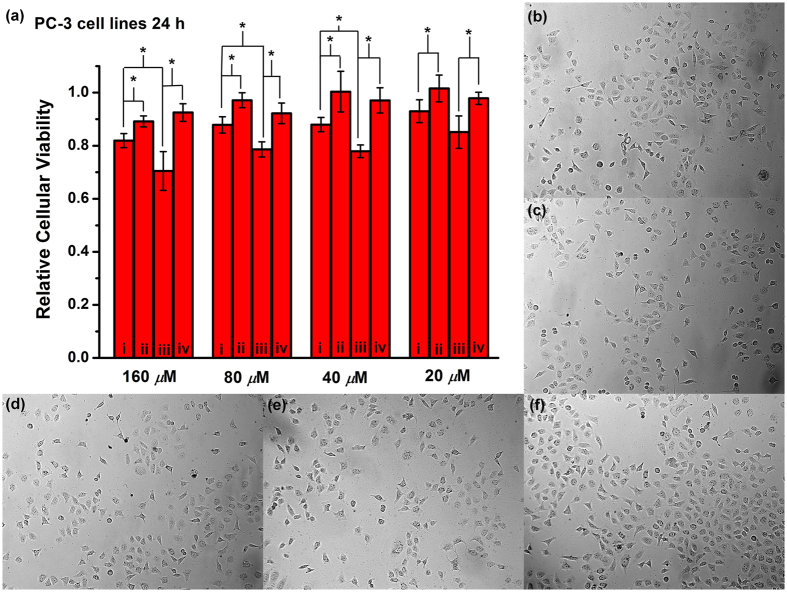
Cytotoxicity experiments of PC-3 cells after 24 h. (**a**) Relative cellular viabilities of PC-3 cells upon the addition of (i) ADA, (ii) ADA + CB[6], (iii) ADA + HACD, and (iv) CB[6] + ADA + HACD at 20‒160 *μ*M after 24 h. Statistically significant differences are indicated with asterisks (*p* < 0.05). Photographs of PC-3 cells: (**b**) blank control and upon the addition of (**c**) ADA, (**d**) ADA + CB[6], (**e**) ADA + HACD, and (**f**) CB[6] + ADA + HACD.

Next, we performed a series of preliminary cytotoxicity experiments to evaluate the safety of the CB[6]‒ADA‒HACD supramolecular complex in PC-3 human prostatic cancer cells prior to the assessment of transfection effects. As shown in Fig. 5, ADA (column i), the ADA‒CB[6] complex (column ii), the ADA‒HACD complex (column iii), and the CB[6]‒ADA‒HACD complex (column iv) at concentrations varying from 20 to 160 *μ*M were added to PC-3 cancer cells. After incubation for 24 h, ADA exhibited considerable cytotoxicity at 160 *μ*M due to the positive charge of the diammonium sidechains; the relative cellular viability was 82%. This viability increased to 93% when the ADA concentration decreased to 20 *μ*M. Because of the protection effect of CB[6], the toxicity of ADA was reduced upon the addition of CB[6]; significantly higher relative cellular viabilities of 90−100% were achieved. Conversely, the supramolecular complex ADA‒HACD showed more intense cytotoxicity than ADA; a relative cellular viability of 70% was observed at 160 *μ*M. This was attributable to the HA receptor-mediated internalization of ADA‒HACD into cancer cells and the increased ADA concentration at the cytomembrane and cytoplasm of PC-3 cancer cells. Intriguingly, the CB[6]‒ADA‒HACD complex showed almost negligible cytotoxicity in PC-3 cells; the relative cellular viabilities were 93‒98%. The morphological characteristics of PC-3 cells were also consistent with results obtained from the cytotoxicity experiments (Fig. 5b‒f). In addition, although relatively higher cytotoxicity was observed for 48 h, the protection effect of CB[6] and the non-toxicity of the CB[6]‒ADA‒HACD complex toward the target cell could be verified by the moderate viability percentages at relatively lower concentration (see Supplementary Fig. S36). These results allow us to conclude that the CB[6]‒ADA‒HACD complex is essentially nontoxic to target cells. Hence, the concentration of the ternary complex used for transfection experiments was fixed at 17 *μ*M; no appreciable side effect was observed in target cells at this concentration.

Although the degree of protonation in ADA could dramatically increase by CB-induced p*K*_a_ shift, the ADA−CB[6] complex was less cytotoxic than ADA alone. These findings may be jointly attributed to the charge distribution and steric stabilization of the cationic diammonium sidechains in ADA by CB[6]. That is, the complexation with CB[6] can greatly delocalize the intensive positive charges of ADA through the N^+^···O^δ−^ ion−dipole interconnection working at ammonium sites in ADA and carbonyl groups in CB[6], which is frequently observed in the crystalline complexes between CB and cationic molecules^51^. Moreover, CB[6] also acts as a sterically demanding component surrounding the positively charged centers to make the system more rigid. This structural feature could prohibit the close location between oppositely charged ionic components, which may allow the dissociation of siRNA at the delivery site. In comparison, the isolated positive charges without the protection of CB[6] in ADA led to the relatively high cytotoxicity.

**Figure 6:**
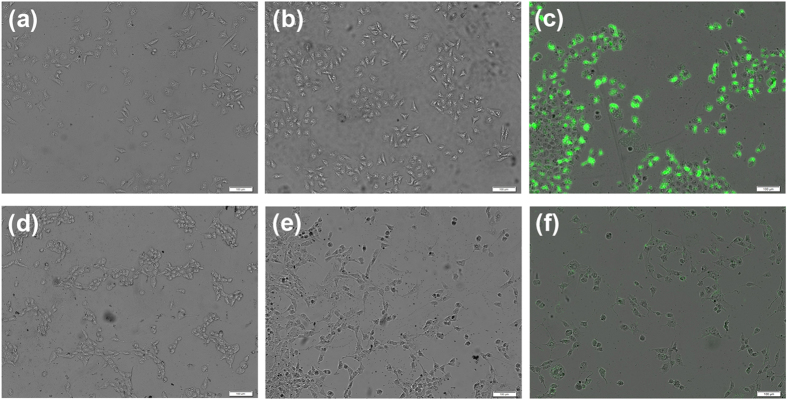
Selective uptake of FAM-siRNA by the supramolecular complexes and assembly. Fluorescence microscopic images of (**a–c**) PC-3 and (**d–f**) NIH3T3 cells incubated with FAM-siRNA containing (**a**,**d**) medium, (**b**,**e**) the ADA‒HACD complex, and (**c,f**) the CB[6]‒ADA‒HACD assembly for 5 h.

To further validate the enhancement of the siRNA binding process by supramolecular p*K*_a_ shift, the uptake of the complex into target cancer cells was studied by confocal fluorescence microscopy using 6-carboxy-fluorescein phosphoramidate (FAM)-labeled siRNA. As shown in Fig. 6a,d, no fluorescence was detected in normal fibroblast NIH3T3 cells or in human prostate PC-3 cancer cells. This finding indicated that free FAM-siRNA cannot enter into cells without the assistance of the CB[6]‒ADA‒HACD complex added as a nanocarrier. Moreover, no detectable fluorescence was observed in these two cell lines after mixing with the ADA‒HACD complex (Fig. 6b,e). In the presence of CB[6], only faint fluorescence was detected in NIH3T3 cells; bright green fluorescence was observed in PC-3 cells under the same experimental conditions (Fig. 6c,f). These results clearly reveal that FAM-siRNA is delivered into cancer cells by receptor-mediated internalization. More importantly, CB[6] plays a primary role in promoting the RNA-binding process in the CB[6]‒ADA‒HACD system.

### Evaluation of EGFP gene silencing effect

**Figure 7:**
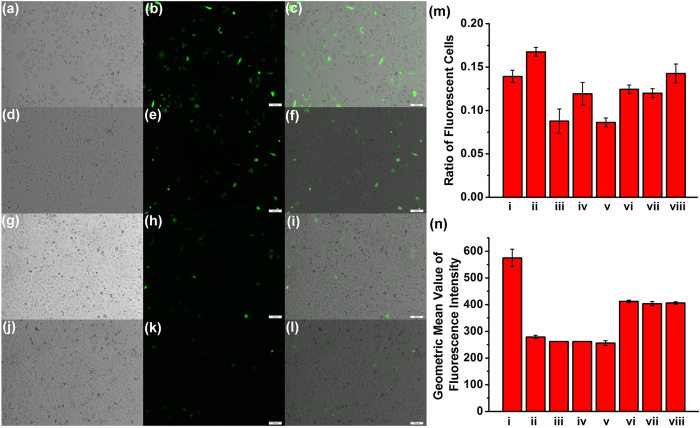
Exogenous EGFP gene silencing effect. Bright-field, fluorescence, and merged images of PC-3 cells after treatment with (**a–c**) Lipofectamine 2000 + EGFP-pDNA (negative control); and Lipofectamine 2000 + EGFP-pDNA followed by (**d–f**) EGFP-siRNA, (**g–i**) Lipofectamine 2000 + EGFP-siRNA, (**j–l**) CB[6]‒ADA‒HACD + EGFP-siRNA (experimental group), respectively. (**m**) Ratios and (**n**) geometric mean values of fluorescence intensity of fluorescent PC-3 cells according to the results of the FCM experiments: (i) negative control; and Lipofectamine 2000 + EGFP-pDNA followed by (ii) Lipofectamine 2000 + EGFP-siRNA, (iii) Lipofectamine RNAiMAX + EGFP-siRNA, (iv) X-tremeGENE + EGFP-siRNA, (v) experimental group, (vi) CB[6]‒ADA‒HACD + HA + EGFP-siRNA, (vii) CB[6]‒ADA + EGFP-siRNA, and (viii) HACD‒ADA + EGFP-siRNA, respectively.

As discussed above, the supramolecular CB[6]‒ADA‒HACD assembly can tightly bind to siRNA to form compact nanoparticles. Having confirmed that these nanoparticles are non-cytotoxic and can be internalized specifically into cancer cells, we then performed exogenetic EGFP (enhanced green fluorescent protein) gene silencing experiments to evaluate the siRNA transfection efficiency of the nanoparticles *in vitro* (see Supplementary Fig. S37). PC-3 cells were first treated with Lipofectamine 2000 and EGFP-pDNA complex as a negative control. Such treatment would efficiently introduce the EGFP gene into cells, resulting in cells expressing green fluorescent protein. Then, the EGFP-siRNA bound by some conventional transfection reagents, ADA-containing binary complexes, and CB[6]‒ADA‒HACD assembly was added to PC-3 cells, respectively. As shown in Fig. 7a‒f, PC-3 cells emitted bright green fluorescence due to transfection with EGFP-pDNA by Lipofectamine 2000. Treatment with free EGFP-siRNA alone did not result in EGFP-silencing in PC-3 cells; free siRNA cannot be internalized into cells and is easily degraded by ribonuclease (RNase) enzymes^52^. However, EGFP-siRNA complexed with Lipofectamine 2000 conferred moderate EGFP-gene silencing effects; the number of cells emitting green fluorescence and the fluorescence intensity of each cell was simultaneously reduced. In contrast, the CB[6]‒ADA‒HACD assembly with EGFP-siRNA conferred a greater EGFP-gene silencing effect in PC-3 cells with enhanced global quenching than that observed in the control groups (Fig. 7g‒l).

The gene knockdown level was also determined quantitatively by flow cytometry (FCM). As shown in Fig. 7m,n, although the number of fluorescent cells was comparable to that of the negative control, the fluorescence intensity sharply decreased after treatment with Lipofectamine 2000 + EGFP-siRNA. These results are indicative of the moderate gene delivery efficiency of Lipofectamine 2000. Moreover, the ADA‒HACD complex could not exhibit obvious gene transfection efficiency under the same experimental conditions. In particular, when an excess amount of HA polymer was used to block the receptors on the PC-3 cancer cell surface or the targeting unit HACD was removed in the ADA‒CB[6] complex, it is found that the nanocarrier lost its original cell recognition ability, thus resulting in a relatively lower gene transfection efficiency. These results further verify the HA receptor-targeted internalization process in cancer cells. Therefore, benefiting from both CB[6] enhanced siRNA binding capability and targeted internalization in cancer cells, our supramolecular nanoparticle composed of CB[6]‒ADA‒HACD and EGFP-siRNA exhibited a greater gene knockdown efficiency and a reduced fluorescence intensity in PC-3 cells, which resulted in a gene delivery capacity comparable to the commercial transfection reagent Lipofectamine RNAiMAX, but much greater than Lipofectamine 2000 and X-tremeGENE. Taken together, these results suggest that the CB[6]‒ADA‒HACD ternary assembly possesses relatively high gene transfection efficacy and can maintain high cell viability simultaneously.

## Conclusions

In conclusion, for the first time, by implementing the supramolecular p*K*_a_ shift concept, we successfully constructed a controlled, targeted and biocompatible nucleic acid binding system, which was achieved by a combined strategy using a supramolecular p*K*_a_ shift and selective self-assembly to realize an efficient nanoparticulate formation using a triad guest ADA with one adamantyl head group and two diamine tails. The adamantyl head group was intended to be selectively bound to *β*-CD, while the diamine tails were to be bound to CB[6]. ^1^H NMR spectral examinations of a solution containing ADA, *β*-CD, and CB[6] revealed the exclusive formation of a *β*-CD‒ADA‒CB[6] ternary complex. Further assembly with *β*-CD-modified HA endowed the ability to target cancer cells to this macrocycle-based nanoarchitecture. The noncovalent entrapment of the diamine moiety of ADA with CB[6] enhanced the binding affinity to siRNA by a macrocycle-induced p*K*_a_ shift, which eventually resulted in siRNA condensation into nanospheres through electrostatic attraction. In cellular experiments, the resulting assembly functioned as a supramolecular non-viral vector in targeted siRNA binding and interference. We also envision that in addition to the enhancement of drug bioavailability, the concept of complexation-induced p*K*_a_ shifts can be applied not only to small drug and dye molecules but also to biofunctional polyamines. This straightforward method provides an attractive and unique tool for controlling the capture and release of other pharmaceutical nucleic acids. Further comparative studies of complexation with various amines are currently in progress.

## Methods

### Reagents

All chemical reagents were commercially available unless noted otherwise. The *β*-CD-appended HA was synthesized according to the reported procedures^32^. NMR data were recorded on 300 and 400 MHz spectrometers. TEM images were acquired using a transmission electron microscope operating at an accelerating voltage of 200 kV. The sample for TEM measurements was prepared by dropping a sample solution onto a copper grid. The grid was then air-dried. AFM experiments were performed in tapping mode in air at room temperature. All ^1^H NMR chemical shifts were referenced to the internal signal of acetone at 2.22 ppm or acetonitrile at 2.06 ppm^53^. For cellular experiments, the conventional transfection reagents (i.e., Lipofectamine 2000, Lipofectamine RNAiMAX, and X-tremeGENE) were purchased from commercial resources. Negative control siRNA (NC-siRNA) and fluorescence labeled FAM-siRNA of the antisense strand 5′-ACGUGACACGUUCGGAGAATT-3′ were purchased from GenePharma Co. Ltd. (Shanghai, China), EGFP-siRNA of the target sequence 5′-GCAAGCTGACCCTGAAGTTC–3′ from Dharmacon, and Hs_FKBP5_5 siRNA of the antisense strand 5′-AUGCUCAAUCUGUUUACCCGT-3′ from QIAGEN. The statistical analysis of the data was carried out using the Student’s t test. Differences were considered statistically significant if the *p* value was < 0.05.

### p*K*_a_ measurement by potentiometric titration

The p*K*_a_ values were determined by using the following equation (1):


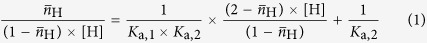


where *K*_a,1_ and *K*_a,2_ are the acidity constants for dissociation of the first and second protons, respectively, and while 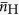 is defined as the mean number of bounded protons and calculated by using the following equation (2):


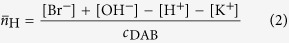


where *c*_DAB_ is the total concentration of investigated DAB; [Br^*−*^] is the concentrations of bromide ion in DAB, [OH^*−*^] is the concentration of hydroxyl ions in aqueous solution, [H^+^] is the concentration of the free hydronium ions determined by pH measurement, and [K^+^] is the concentration of added KOH, respectively. Accordingly, the species distribution of doubly protonated DAB (H_2_DAB) at the physiological pH (7.20) could be calculated by the following equation (3)^54^:


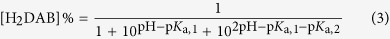


### Association constant (*K*_a_) determination

The association constant (*K*_a_) for a stoichiometric 1:1 complex of ADA with native *β*-CD was calculated by using the non-linear least-squares fit of the titration data to the following equation (4)^55^:


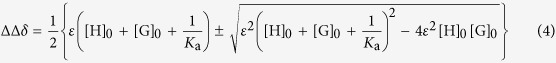


where ΔΔ*δ* is the NMR chemical shift change of ADA upon addition of native *β*-CD and defined as ∆∆*δ* = ∆*δ* (with *β*-CD) −∆*δ* (without *β*-CD), *ε* is the sensitivity factor, and [H]_0_ and [G]_0_ are the initial concentrations of native *β*-CD and ADA, respectively.

### Isothermal titration calorimetry (ITC) measurements

The ITC experiments were performed by an isothermal titration microcalorimeter at atmospheric pressure and at 25.00 °C in aqueous solution, giving the association constants (*K*_a_) and the thermodynamic parameters of DAB upon complexation with CB[6]. A solution of CB[6] (1.868 mM) in a 0.250 mL syringe was sequentially injected with stirring at 300 rpm into a solution of DAB (0.1744 mM) in the sample cell (1.4227 mL volume). All the thermodynamic parameters reported in this work were obtained by using the ‘one set of binding sites’ model. Two independent titration experiments were performed to afford self-consistent parameters and to give the averaged values.

### Agarose gel electrophoresis assay

Agarose gel electrophoresis experiments were performed in TAE buffer (0.04 M Tris, 0.02 M acetic acid, and 2.0 mM ethylenediaminetetraacetic acid (EDTA)) at 25 °C. Following electrophoresis, siRNA bands were stained in an ethidium bromide (EB) solution and were visualized under UV light at 302 nm. The condensation ability of the resulting complexes and assemblies to siRNA was measured by analyzing the electrophoretic mobility at different N/P ratios on agarose gel. In the electrophoresis assay, the N/P ratio is defined as the ratio of positively charged ammoniums in ADA to negatively charged phosphates in siRNA; the molar ratio of CB[6] to ADA was fixed at 2:1. Gene transfection and cytotoxicity studies were performed at N/P = 20, the optimal condition for siRNA condensation, as determined in Supplementary Fig. S34.

### Cytotoxicity experiments

PC-3 human prostatic cancer cells were cultured for 24 h in RPMI-1640 medium, which was supplemented with 10% fetal bovine serum (FBS), in 96-well plates (5 × 10^4^ cells mL^−1^, 100 *μ*L per well). The cells were incubated with ADA, ADA‒CB[6], ADA‒HACD, and CB[6]‒ADA‒HACD complexes at different concentrations; [ADA] = 20, 40, 80, and 160 *μ*M and [CB[6]] = 2[ADA] = 34[HACD]. After incubation for 24 and 48 h, the relative cellular viability was determined by the MTT (3-(4,5-dimethylthiazol-2-yl)-2,5-diphenyltetrazolium bromide) assay. All data are presented as the mean ± standard deviation.

In the comparative study, the PC-3 human prostatic cancer cells were incubated with 1 equiv ternary assembly ([CB[6]] = 2[ADA] = 34[HACD] = 34 *μ*M), 10 equiv ternary assembly ([CB[6]] = 2[ADA] = 34[HACD] = 340 *μ*M), 1 equiv Lipofectamine 2000 (1.5 *μ*L in 1 mL 1640 medium), and 10 equiv Lipofectamine 2000 (15 *μ*L in 1 mL 1640 medium), respectively. After incubation for 24 h, the relative cellular viability was measured with MTT assay. All the loading concentrations of the commercial transfection reagents were used according to the manufacturer’s instruction. All data are presented as the mean ± standard deviation (see Supplementary Fig. S35).

### Fluorescence imaging experiments

PC-3 cells were cultured in RPMI-1640 medium, and NIH3T3 mouse embryonic fibroblasts were cultured for 24 h in Dulbecco’s modified Eagle’s medium (DMEM), which were supplemented with 10% FBS, in 6-well plates (1 × 10^5^ cells mL^−1^, 2 mL per well). Then, CB[6]‒ADA‒HACD assembly ([CB[6]] = 2[ADA] = 34[HACD] = 34 *μ*M) with FAM-labeled NC-siRNA (80 nM, N/P = 20) was added to the culture medium. After incubation for 5 h, the medium was discarded, the cells were washed by fresh cold PBS, and then the fresh medium was added. The cells were examined by fluorescence microscopy.

### EGFP gene silencing experiments

PC-3 cells were cultured for 24 h in RPMI-1640 medium, which was supplemented with 10% FBS, in 6-well plates (2 × 10^5^ cells mL^−1^, 2 mL per well). Then, the culture medium was removed and the FBS-free 1640 medium containing Lipofectamine 2000 (2 *μ*L per 1 mL 1640 medium) and EGFP-pDNA (1.9 *μ*g per 1 mL 1640 medium) was added to the culture plates. After incubation for 3 h, the medium was discarded and full component 1640 medium (containing FBS), full component 1640 medium containing EGFP-siRNA (80 nM), FBS-free 1640 medium containing Lipofectamine 2000 (1.5 *μ*L per 1 mL 1640 medium) and EGFP-siRNA (80 nM) which was replaced by fresh full component 1640 medium after another 4 h, and full component 1640 medium containing CB[6]‒ADA‒HACD ([CB[6]] = 2[ADA] = 34[HACD] = 34 *μ*M) and EGFP-siRNA (80 nM, N/P = 20) were added to the culture plates. After incubation for another 24 h, the cells were examined by fluorescence microscope and then dissociated by trypsin for FCM measurements.

## Additional Information

**How to cite this article**: Zhang, Y.-M. *et al*. Polysaccharide Nanoparticles for Efficient siRNA Targeting in Cancer Cells by Supramolecular p*K*_a_ Shift. *Sci. Rep.* **6**, 28848; doi: 10.1038/srep28848 (2016).

## Supplementary Material

Supplementary Information
