# Supplementary material for: Polysaccharide Nanoparticles for Efficient siRNA Targeting in Cancer Cells by Supramolecular pKa Shift
Source: Sci Rep. 2016 Jul 1;6:28848. doi: 10.1038/srep28848 (PMC4929451; doi:10.1038/srep28848)
Supplement: Supplementary Information [file srep28848-s1.doc]

Supplementary Information

Polysaccharide Nanoparticles for Efficient siRNA Targeting in Cancer Cells by Supramolecular p*K*_a_ Shift

Ying-Ming Zhang^1^, Yang Yang^1,3^, Yu-Hui Zhang^1^ & Yu Liu^1,2^

^1^Department of Chemistry, State Key Laboratory of Elemento-Organic Chemistry, Nankai University, Tianjin 300071, P. R. China

^2^Collaborative Innovation Center of Chemical Science and Engineering (Tianjin), Tianjin 300071, P. R. China

^3^Present address: School of Chemical Engineering and Technology, Hebei University of Technology, Tianjin 300130, P. R. China

*Address correspondence to yuliu@nankai.edu.cn

**Synthesis of guest molecule ADA and reference compound DAB.**

**Supplementary Figure S1.** Synthesis of compound **4**.

A mixture of 1-adamantylbromomethyl ketone **2** (0.96 mmol, 247 mg, 1.0 equiv), 3,5-bis(azidomethyl)phenol **3**[[1]](#endnote-2) (1.3 mmol, 265 mg, 1.4 equiv), and potassium carbonate (3.9 mmol, 540 mg, 4.0 equiv) in acetone was heated to reflux overnight. Insoluble precipitates were removed by filtration and the filtrate was evaporated in vacuo. The residue was dissolved in CHCl_3_ and the organic layer was extracted with brine and dried over sodium sulfate. The crude product was further purified by flash column chromatography (silica gel) using petroleum ether (PE)/EtOAc (8:1, v:v) as eluent to give compound **4** as colorless oil (*R*_f_ = 0.7, yield: 71%). ^1^H NMR (400 MHz, CDCl_3_, ppm) *δ* 6.89 (s, 1H), 6.81 (s, 2H), 4.91 (s, 2H), 4.35 (s, 4H), 2.12 (m, 3H), 1.96 (m, 6H), 1.84–1.75 (m, 6H). ^13^C NMR (100 MHz, CDCl_3_, ppm) *δ* 208.5, 158.8, 137.7, 120.5, 114.1, 68.7, 54.4, 45.6, 38.1, 36.4, 28.7. HRMS (ESI): *m*/*z* calcd for C_20_H_24_N_6_O_2_Na^+^ 403.1858 [M + Na]^+^; found: 403.1858.

**Supplementary Figure S2.** Synthesis of compound **5**.

A mixture of **4** (1.94 mmol, 738 mg, 1.0 equiv) and triphenylphosphine (6.0 mmol, 1.56 g, 3.0 equiv) was stirred for 12 h in THF at room temperature. After removal of the solvent in vacuo, the residue was dissolved in EtOAc. White precipitates obtained upon addition of HCl (0.5 mL) were collected and washed with an excess amount of EtOAc to yield compound **5** as white solid (yield: 42%). ^1^H NMR (400 MHz, D_2_O, ppm) *δ* 7.02 (s, 1H), 6.94 (s, 2H), 5.12 (s, 2H), 4.08 (s, 4H), 1.96 (m, 3H), 1.83 (m, 6H), 1.71–1.61 (m, 6H). ^13^C NMR (100 MHz, D_2_O, ppm) *δ* 215.3, 158.0, 135.2, 122.1, 115.7, 69.2, 45.5, 42.6, 37.2, 35.7, 27.3. HRMS (ESI): *m*/*z* calcd for C_20_H_29_N_2_O_2_: 329.2229 [M ‒ 2HCl + H]^+^; found: 329.2225.

**Supplementary Figure S3.** Synthesis route of compound **7**.

A mixture of **5** (0.3 mmol, 120 mg, 1.0 equiv), compound **6**[[2]](#endnote-3) (0.69 mmol, 400 mg, 2.3 equiv), and triethylamine (0.18 mL, 4.0 equiv) was stirred for 20 h in dichloromethane (20 mL) at room temperature. Then, the reaction mixture was extracted with brine and dried over sodium sulfate. After the organic solvent was removed under vacuum, the crude product obtained was subjected to column chromatography with gradient elution from PE/EtOAc (from 2:1, v:v) to EtOAc to afford compound **7** as white solid (*R*_f_ = 0.5 using EtOAc as eluent, yield: 62%). ^1^H NMR (400 MHz, CDCl_3_, ppm) *δ* 7.33‒7.30 (m, 20H), 6.62 (m, 1H), 6.70 (m, 2H), 5.12 (s, 4H), 5.06 (s, 4H), 4.80 (s, 2H), 4.31 (m, 4H), 3.90 (s, 4H). 3.33 (m, 4H), 3.10 (m, 4H), 2.06 (m, 3H), 1.88 (m, 6H), 1.79‒1.69 (m, 6H), 1.53−1.43 (m, 8H). ^13^C NMR (100 MHz, CDCl_3_, ppm) *δ*. 210.0, 170.1, 159.2, 157.3, 141.0, 137.5, 137.1, 129.3, 129.2, 128.8, 128.7, 128.5, 120.2, 113.3, 69.2, 68.3, 67.2, 49.1, 46.2, 43.7, 41.2, 38.7, 37.1, 28.4, 27.6. HRMS (ESI): *m*/*z* calcd for C_64_H_77_N_6_O_12_ 1121.5599 [M + H]^+^; found: 1121.5598; *m*/*z* calcd for C_64_H_76_N_6_O_12_Na 1143.5419 [M + Na]^+^, found: 1143.5418.

**Fig. S4. Supplementary Figure S4.** Synthesis of guest compound **1** (ADA∙4HBr).

Cbz-protected precursor **7** (0.2 mmol, 212 mg) was added to 33 wt% HBr‒HOAc (5 mL), and the reaction mixture was stirred for 10 h at room temperature. After adding Et_2_O, the precipitates obtained were collected by centrifugation and washed with an excess amount of Et_2_O. The target compound **1** (ADA∙4HBr) was quantitatively obtained as white solid. ^1^H NMR (400 MHz, D_2_O, ppm) *δ* 6.66 (s, 1H), 6.51 (s, 2H), 4.94 (s, 2H), 4.15 (s, 4H), 3.69 (s, 4H), 2.88 (m, 4H), 2.78 (m, 4H). 1.82 (m, 3H), 1.68 (m, 6H), 1.54 (m, 14H). ^13^C NMR (100 MHz, D_2_O, ppm) *δ* 215.5, 165.9, 157.8, 139.8, 119.8, 112.6, 69.3, 48.1, 47.1, 45.6, 42.9, 38.8, 37.3, 35.8, 27.4, 23.9, 22.7. HRMS (ESI): *m*/*z* calcd for C_32_H_54_N_6_O_4_^2+^ 293.2098 [M ‒ 4Br ‒ 2H]^2+^, found: 293.2095; *m*/*z* calcd for C_32_H_51_N_5_O_4_^2+^ 284.6971 [M ‒ 4Br ‒ 2H ‒ NH_3_]^2+^, found: 284.6965; *m*/*z* calcd for C_32_H_48_N_4_O_4_^2+^ 276.1838 [M ‒ 4Br ‒ 2H ‒ 2NH_3_]^2+^, found: 276.1832.

**Supplementary Figure S5.** Synthesis route of compound **8**.

The synthesis of compound **8** is similar to compound **7** by using benzylamine hydrochloride as the starting material (*R*_f_ = 0.6 using PE/EtOAc (3:1, v:v) as eluent, yield: 60%). ^1^H NMR (400 MHz, CDCl_3_, ppm) *δ* 7.35‒7.27 (m, 15H), 5.12‒5.08 (m, 4H), 4.40 (m, 2H), 3.92 (s, 2H), 3.36‒3.33 (m, 2H), 3.11 (m, 2H), 1.53‒1.41 (m, 4H). ^13^C NMR (100 MHz, CDCl_3_, ppm) *δ*. 155.4, 135.6, 135.1, 127.7, 127.6, 127.5, 127.3, 127.1, 127.0, 126.6, 66.7, 65.6, 47.3, 42.4, 39.5, 25.9. HRMS (ESI): *m*/*z* calcd for C_29_H_33_N_3_NaO_5_ 526.2318 [M + Na]^+^; found: 526.2318.

**Supplementary Figure S6.** Synthesis of reference compound DAB.

The synthesis of DAB is similar to ADA by using the compound **8** as the starting material. ^1^H NMR (400 MHz, D_2_O, ppm) *δ* 7.41‒7.31 (m, 5H), 4.41 (s, 2H), 3.92 (s, 2H), 3.13‒3.09 (m, 3H), 3.01‒2.98 (m, 3H), 1.77‒1.72 (m, 4H). ^13^C NMR (100 MHz, D_2_O, ppm) *δ* 165.9, 137.4,

128.8, 127.7, 127.3, 47.9, 46.9, 43.1, 38.7, 23.8, 22.6. ESI-MS: *m*/*z* calcd for C_13_H_22_N_3_O^+^ 236.18 [M + H]^+^, found: 236.20.


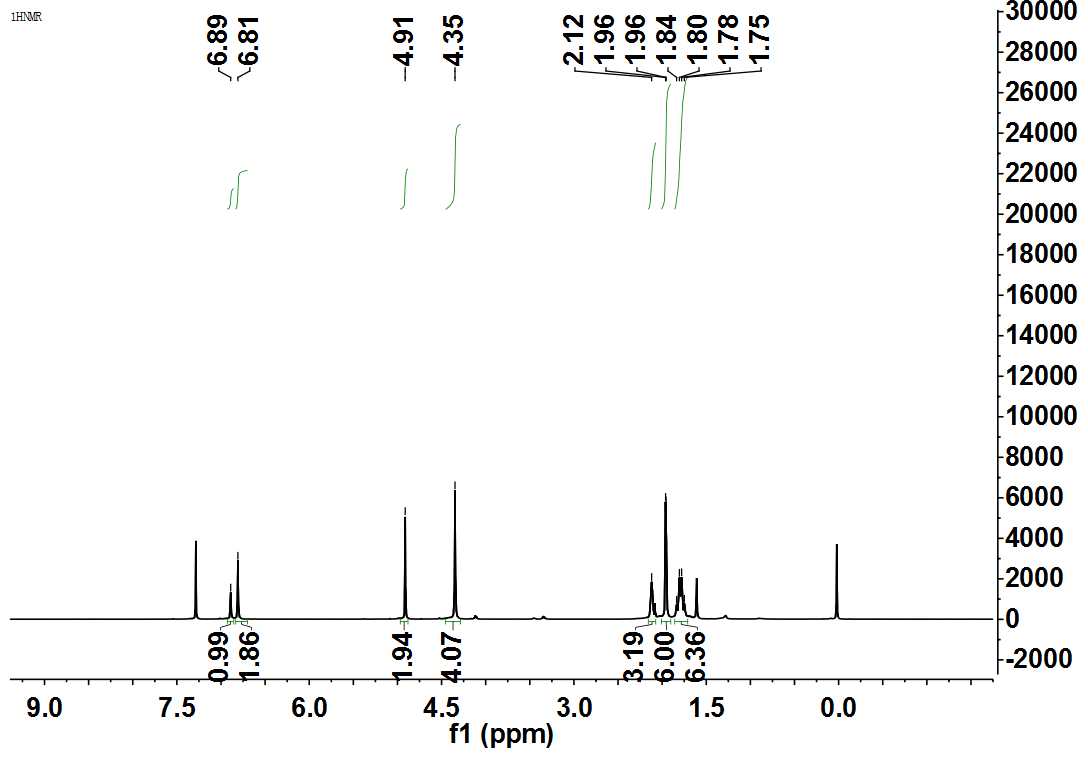


**Supplementary Figure S7.** ^1^H NMR spectrum of compound **4** (400 MHz, CDCl_3_, 25 °C).


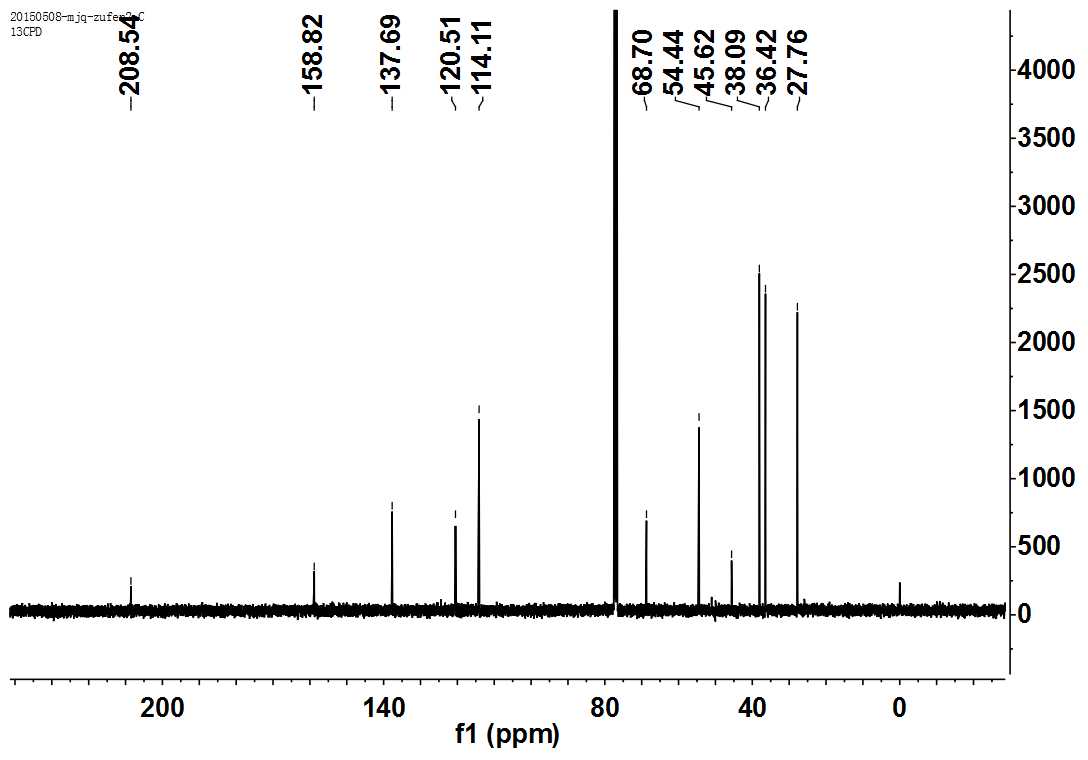


**Supplementary Figure S8.** ^13^C NMR spectrum of compound **4** (100 MHz, CDCl_3_, 25 °C).

**Supplementary Figure S9.** High-resolution ESI mass spectrum of compound **4**. The peak at *m*/*z* 403.1858 corresponds to [M + Na]^+^.


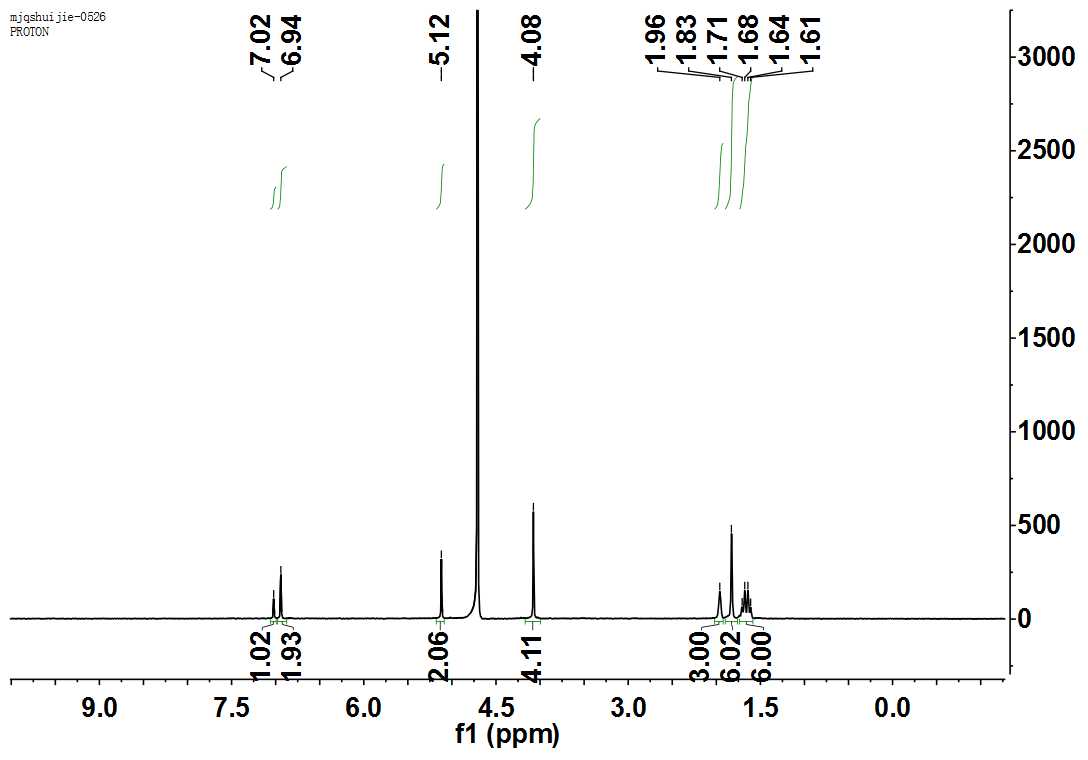


**Supplementary Figure S10.** ^1^H NMR spectrum of compound **5** (400 MHz, D_2_O, 25 °C).


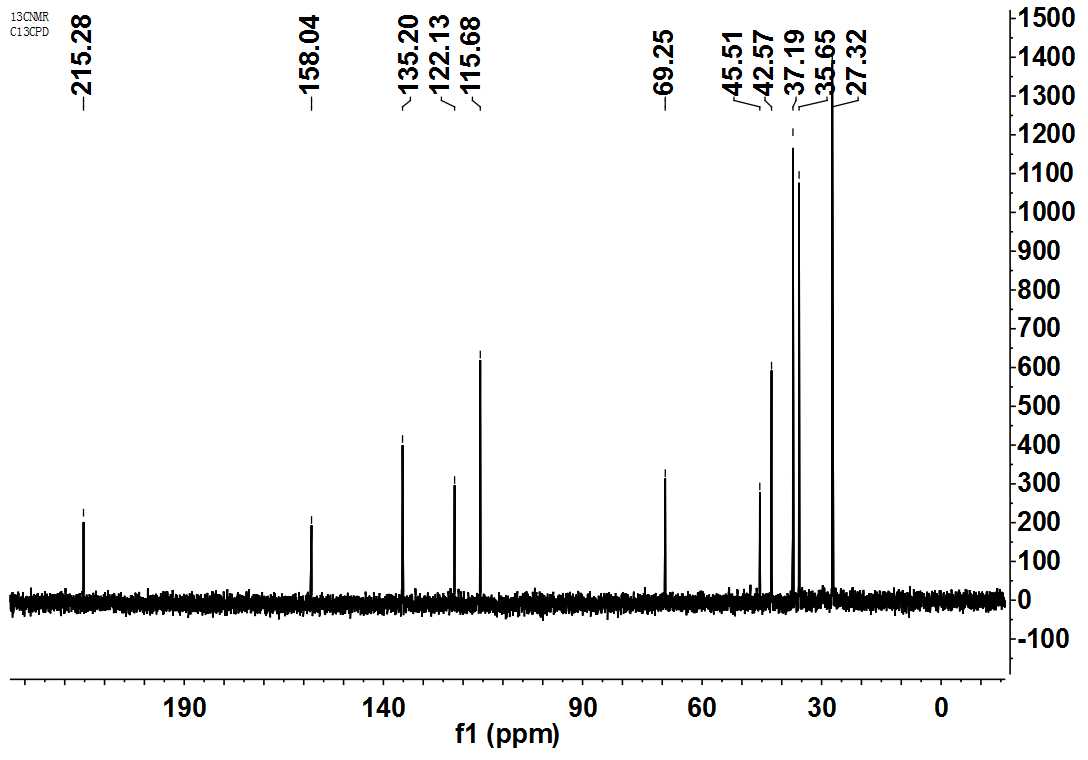


**Supplementary Figure S11.** ^13^C NMR spectrum of compound **5** (100 MHz, D_2_O, 25 °C).

**Supplementary Figure S12.** High-resolution ESI mass spectrum of compound **5**. The peak at *m*/*z* 329.2225 corresponds to [M + H]^+^.


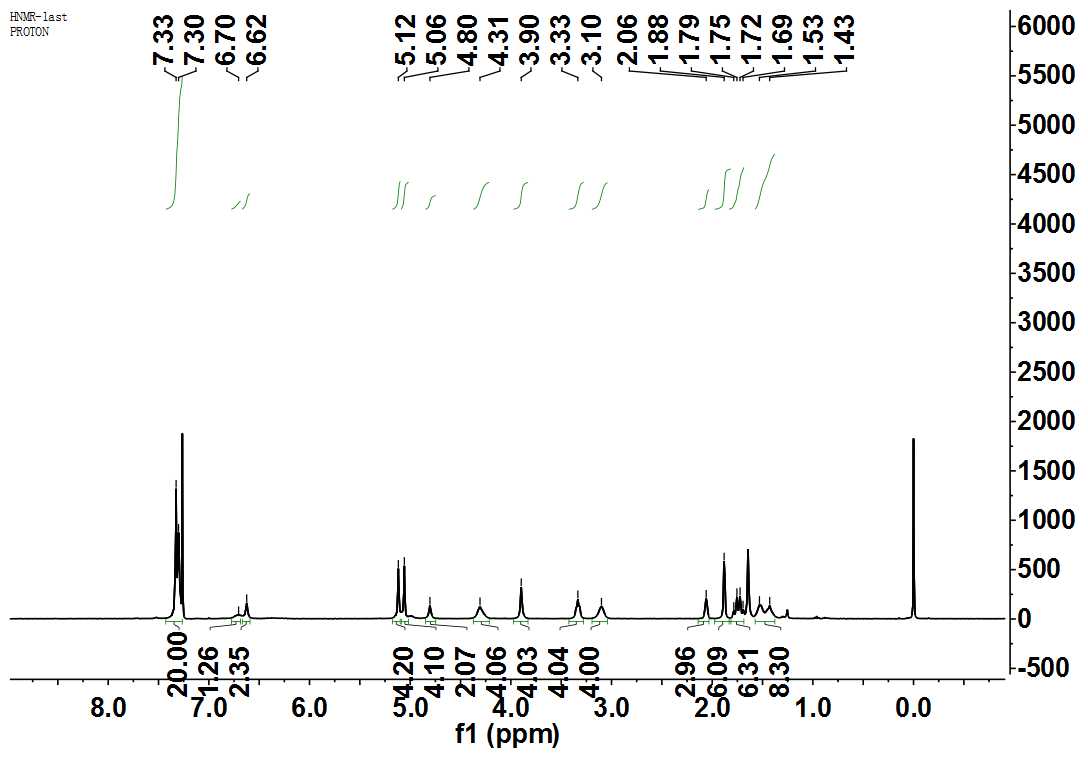


**Supplementary Figure S13.** ^1^H NMR spectrum of compound **7** (400 MHz, CDCl_3_, 25 °C).


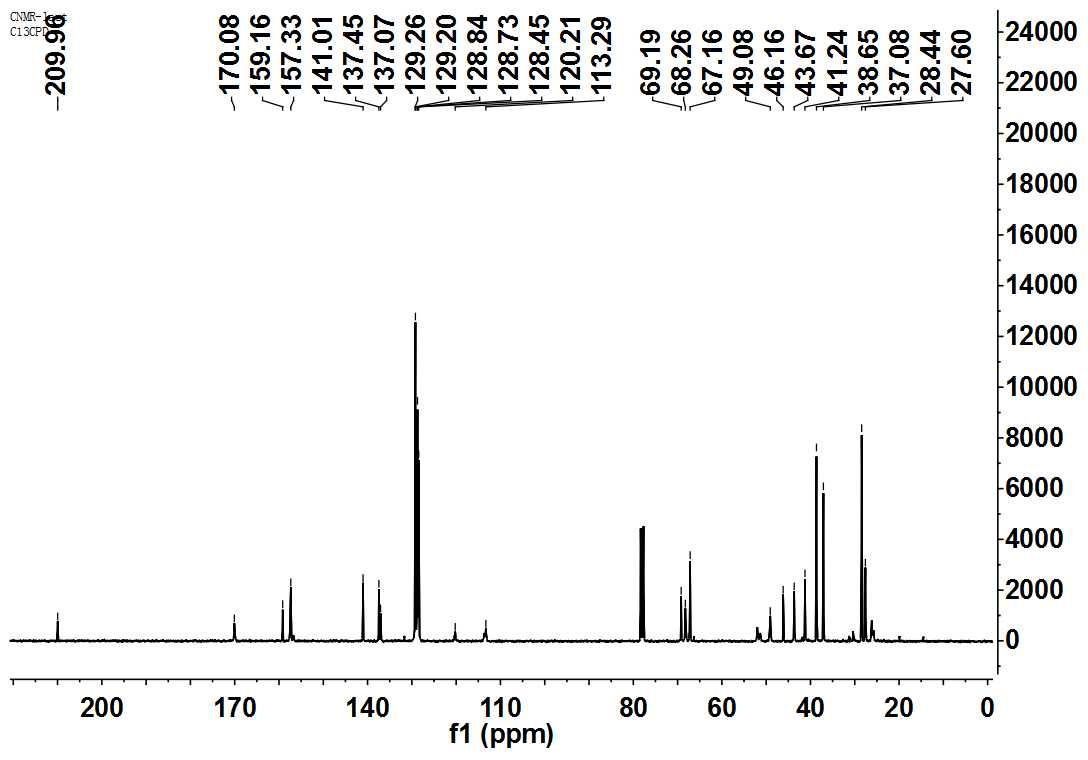


**Supplementary Figure S14.** ^13^C NMR spectrum of compound **7** (100 MHz, CDCl_3_, 25 °C).

**Supplementary Figure S15.** High-resolution ESI mass spectrum of compound **7**. The peaks at *m*/*z* 1121.5598 and 1143.5418 correspond to [M + H]^+^ and [M + Na]^+^, respectively.


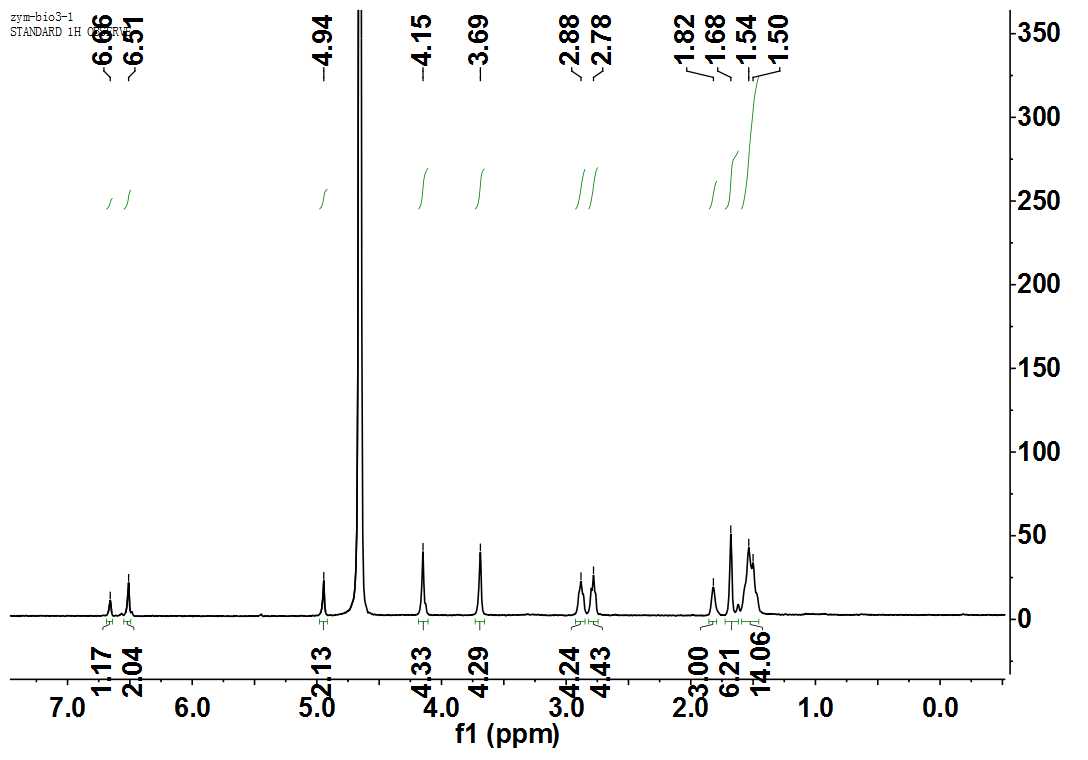


**Supplementary Figure S16.** ^1^H NMR spectrum of compound **1** (400 MHz, D_2_O, 25 °C).


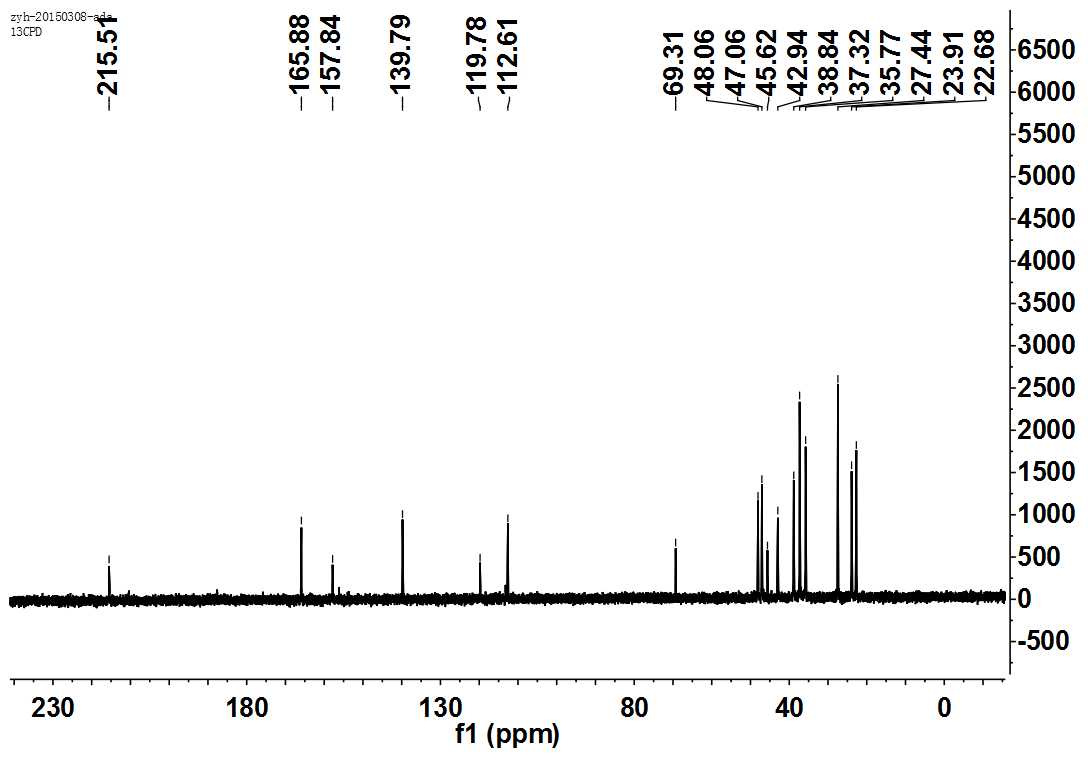


**Supplementary Figure S17.** ^13^C NMR spectrum of compound **1** (100 MHz, D_2_O, 25 °C).

**Supplementary Figure S18.** High-resolution ESI mass spectrum of compound **1** in water. The peak at *m*/*z* 293.2095 corresponds to [ADA + 2H]^2+^.

**Supplementary Figures.**


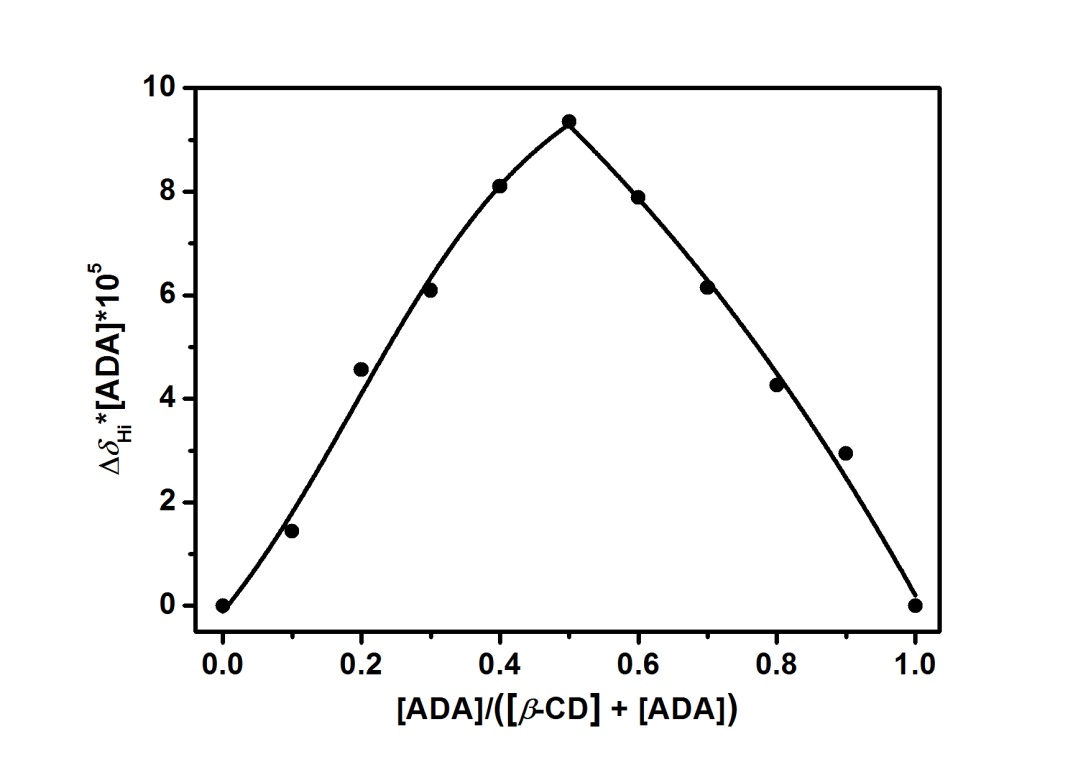


**Supplementary Figure S19.** Job plot for determining the stoichiometry of ADA−*β*-CD complex in D_2_O by monitoring the ^1^H NMR chemical shift changes of ADA’s H_i_ proton ([*β*-CD] + [ADA] = 1.0 mM, 400 MHz, 25 °C).


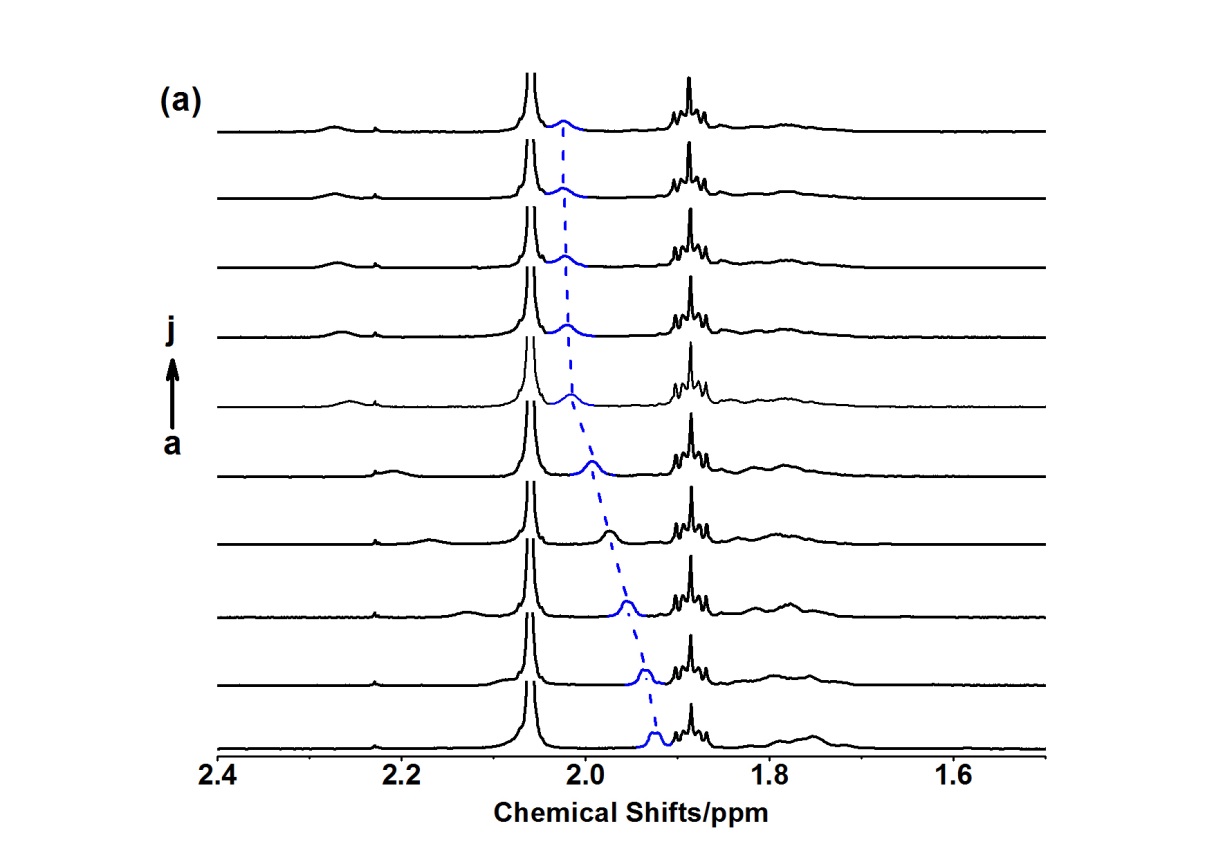


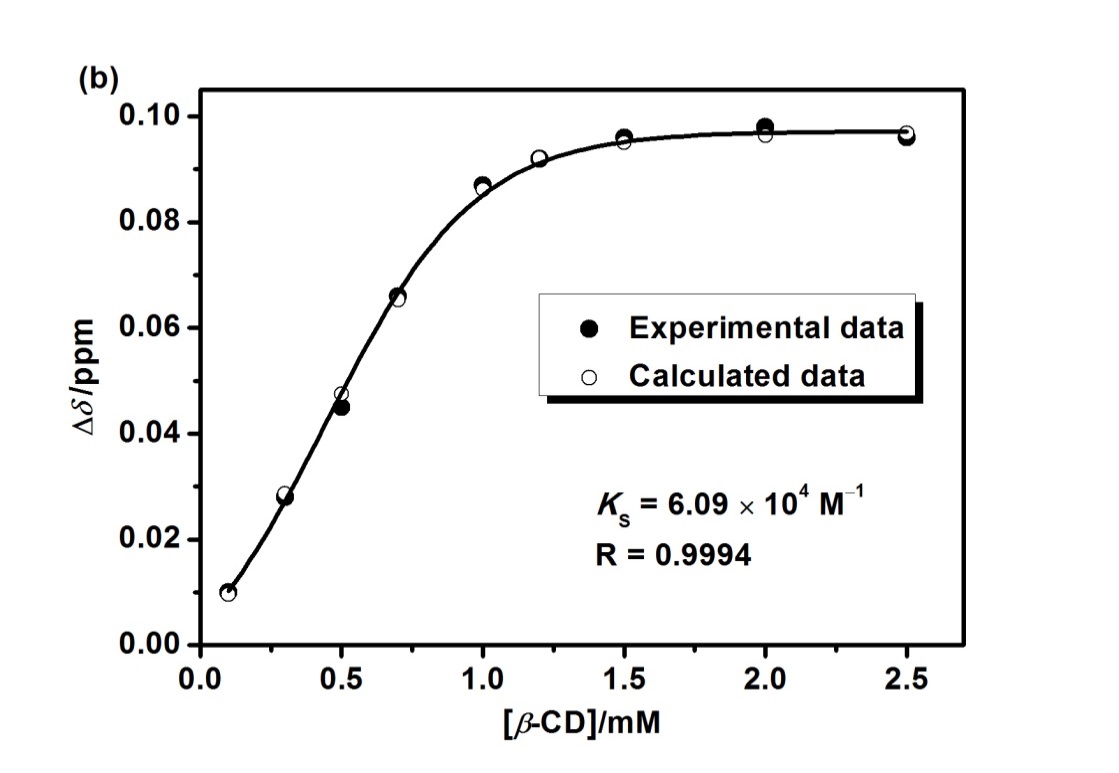


**Supplementary Figure S20.** (a) ^1^H NMR chemical shift changes of ADA’s H_h_ proton upon titration with native *β*-CD in D_2_O containing 0.5% CH_3_CN as internal reference at 25 °C ([ADA] fixed at 1.0 mM; [*β*-CD] varied from 0 to 2.5 mM (line a to j)) and (b) the nonlinear least-squares fit of the chemical shift changes of ADA’s H_h_ proton originally located at *δ* 1.92 ppm to calculate the 1:1 association constant (*K*_a_) of *β*-CD with ADA.


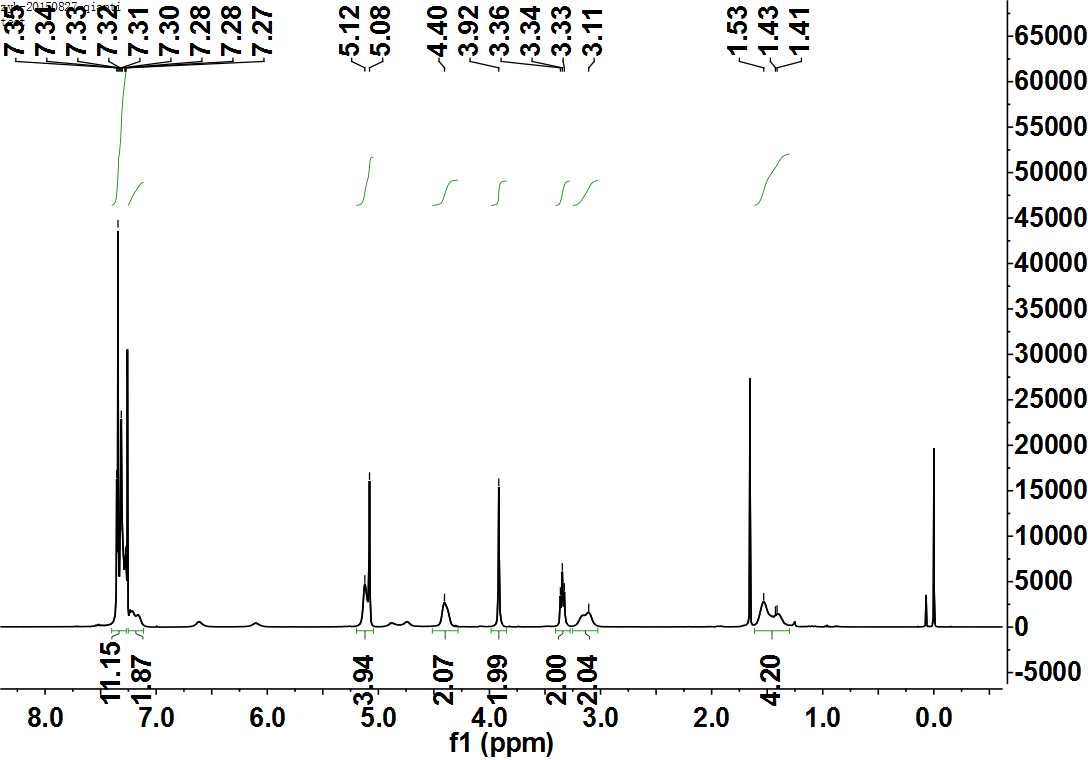


**Supplementary Figure S21.** ^1^H NMR spectrum of compound **8** (400 MHz, CDCl_3_, 25 °C).


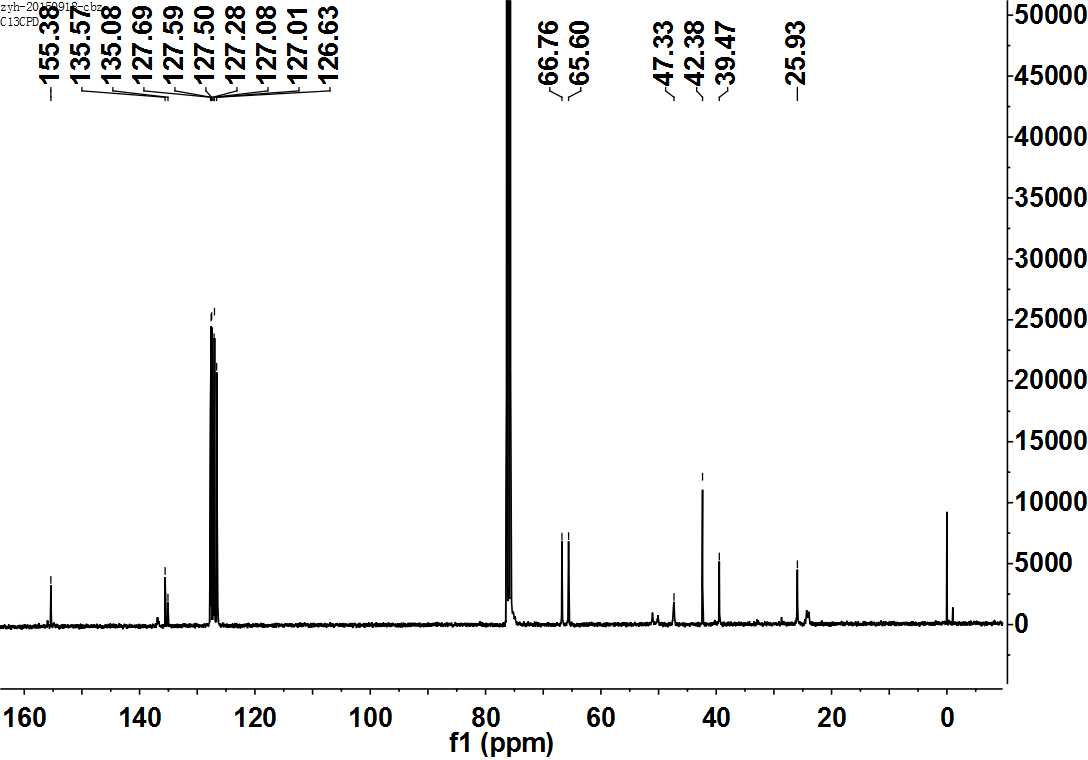


**Supplementary Figure S22.** ^13^C NMR spectrum of compound **8** (100 MHz, CDCl_3_, 25 °C).

**Supplementary Figure S23.** High-resolution ESI mass spectrum of compound **8**. The peak at *m*/*z* 526.2318 corresponds to [M + Na]^+^.


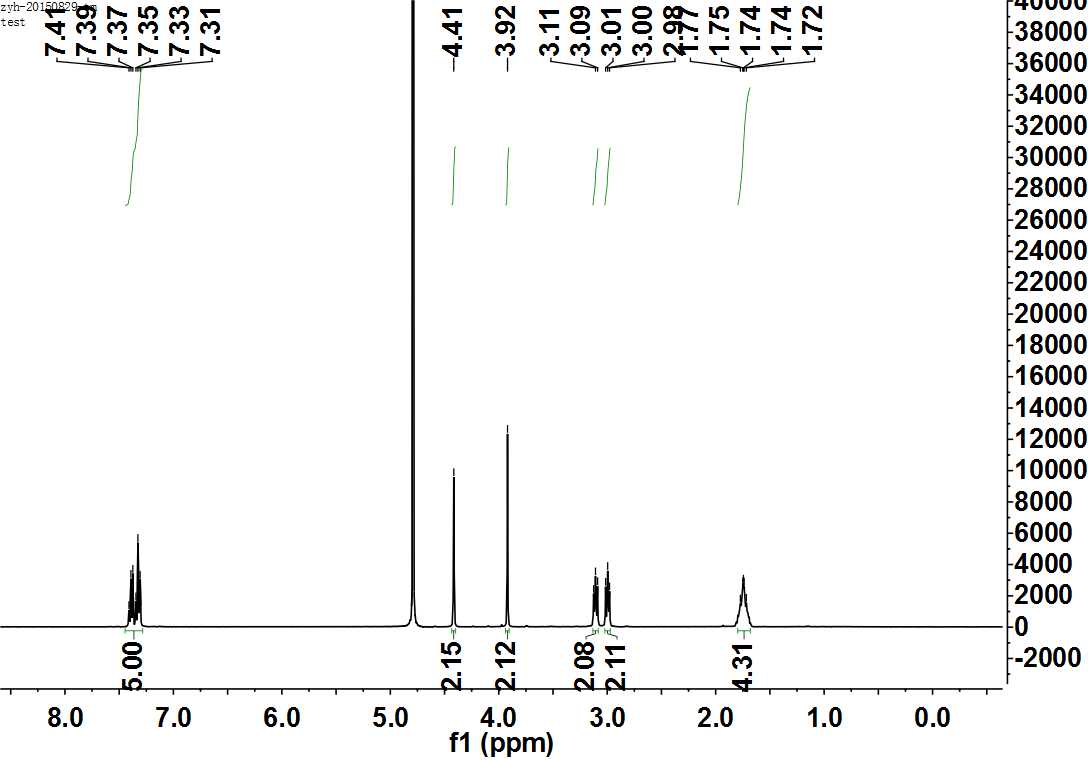


**Supplementary Figure S24.** ^1^H NMR spectrum of DAB (400 MHz, D_2_O, 25 °C).


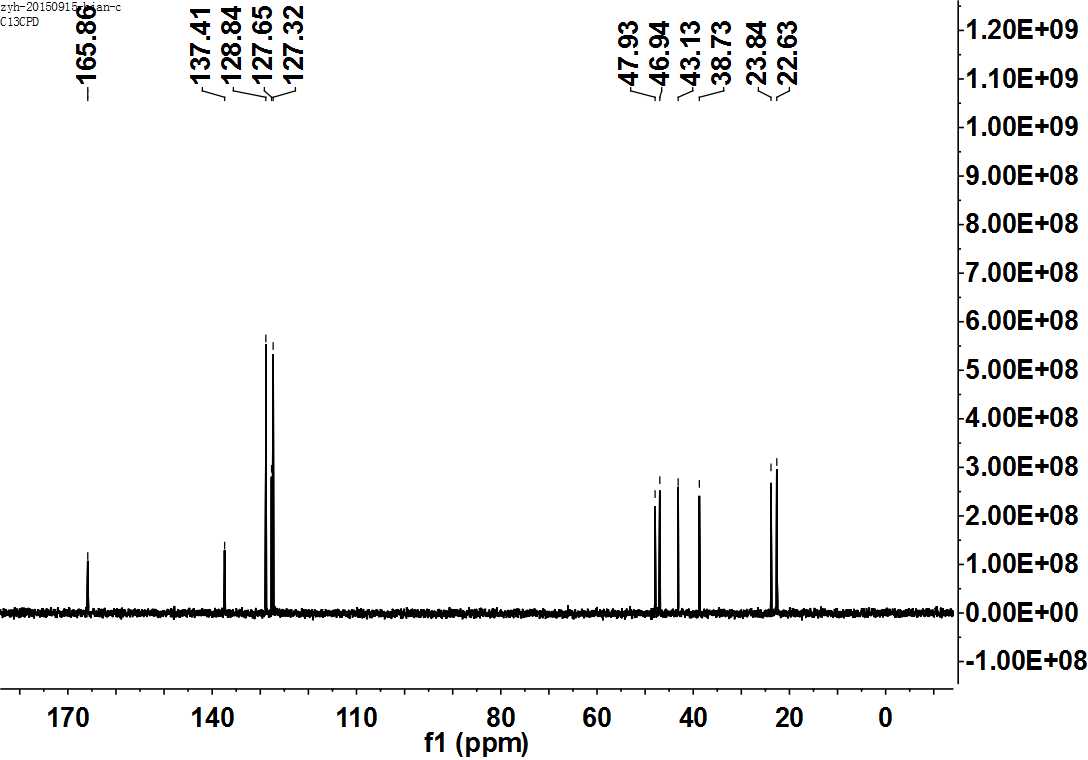


**Supplementary Figure S25.** ^13^C NMR spectrum of DAB (100 MHz, D_2_O, 25 °C).


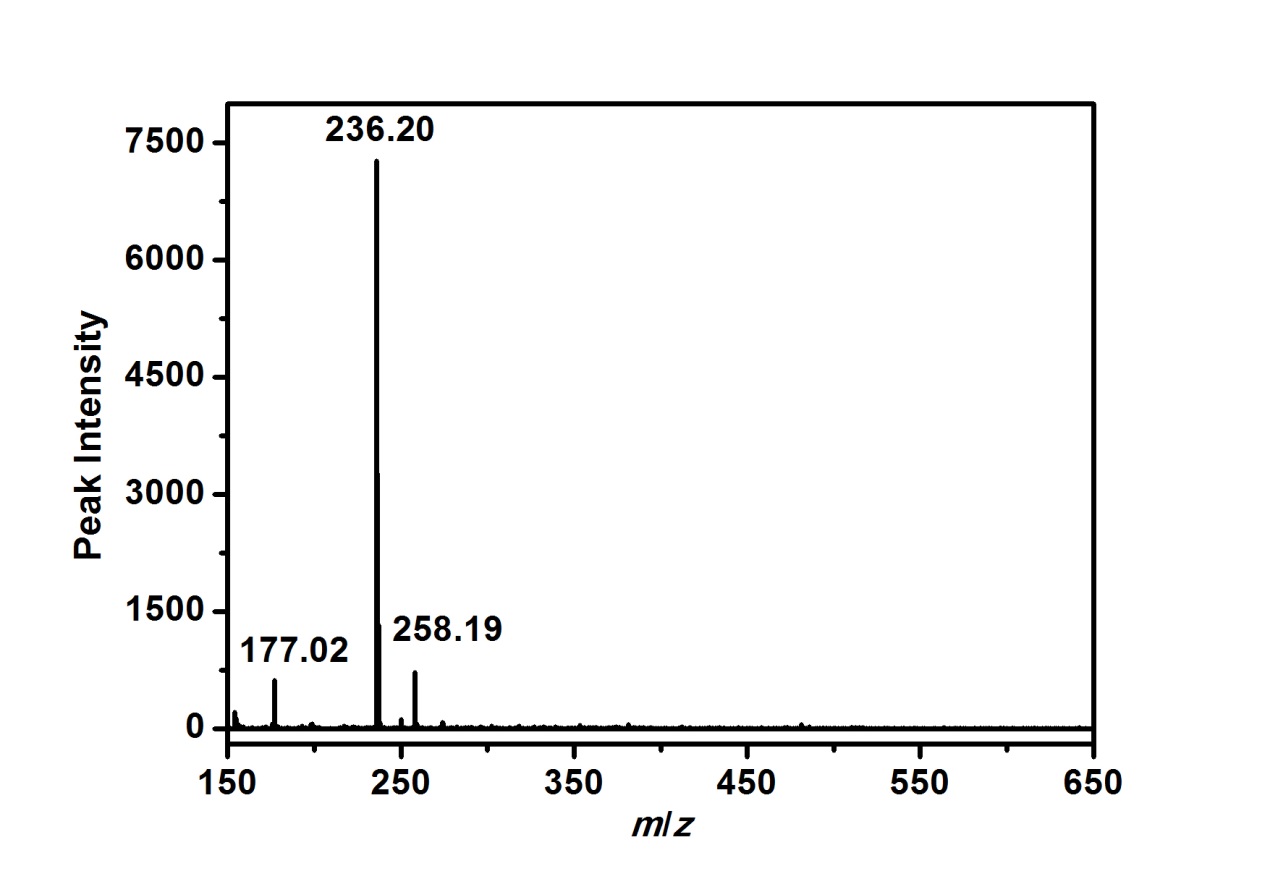


**Supplementary Figure S26.** ESI mass spectrum of DAB. The peak at *m*/*z* 236.20 corresponds to [DAB + H]^+^.


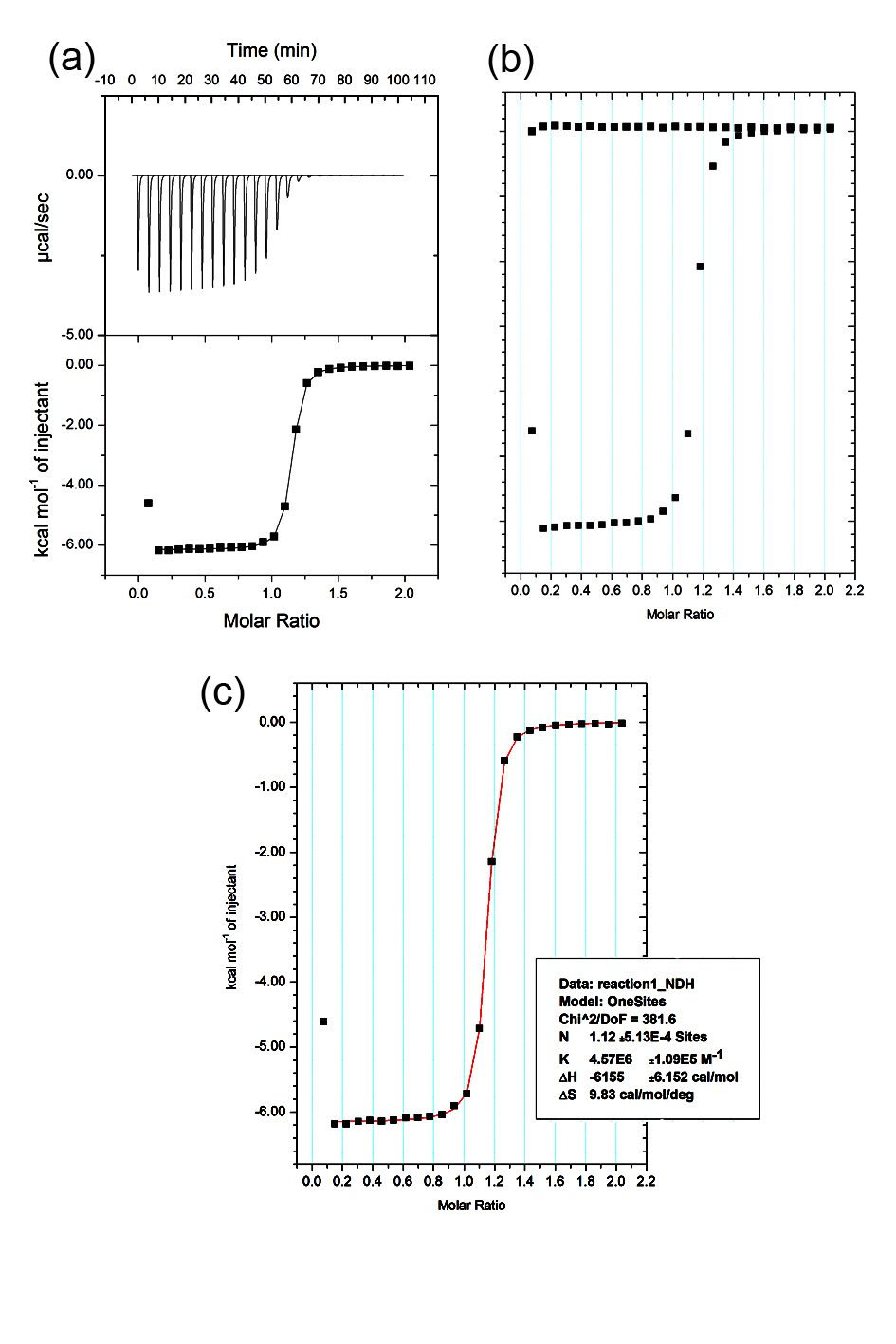


**Supplementary Figure S27.** Calorimetric titrations in phosphate buffer solution (pH = 7.40, *I* = 0.01 M) for sequential 25 injections (10 *μ*L per injection) of CB[6] solution (1.868 mM) injecting into DAB solution (0.1744 mM): (a) raw data and apparent reaction heat; (b) heat effects of the dilution and of the complexation reaction; (c) “Net” heat effects fitted using the “one set of binding sites” model. The thermodynamic data in DAB‒CB[6] complex were obtained as *K*_a_ = (4.51 ± 0.06) × 10^6^ M^‒1^, Δ*G* = ‒37.98 ± 0.03 kJ∙mol^‒1^; Δ*H* = ‒25.92 ± 0.17 kJ∙mol^‒1^; and *T*Δ*S* = 12.06 ± 0.20 kJ∙mol^‒1^, respectively.


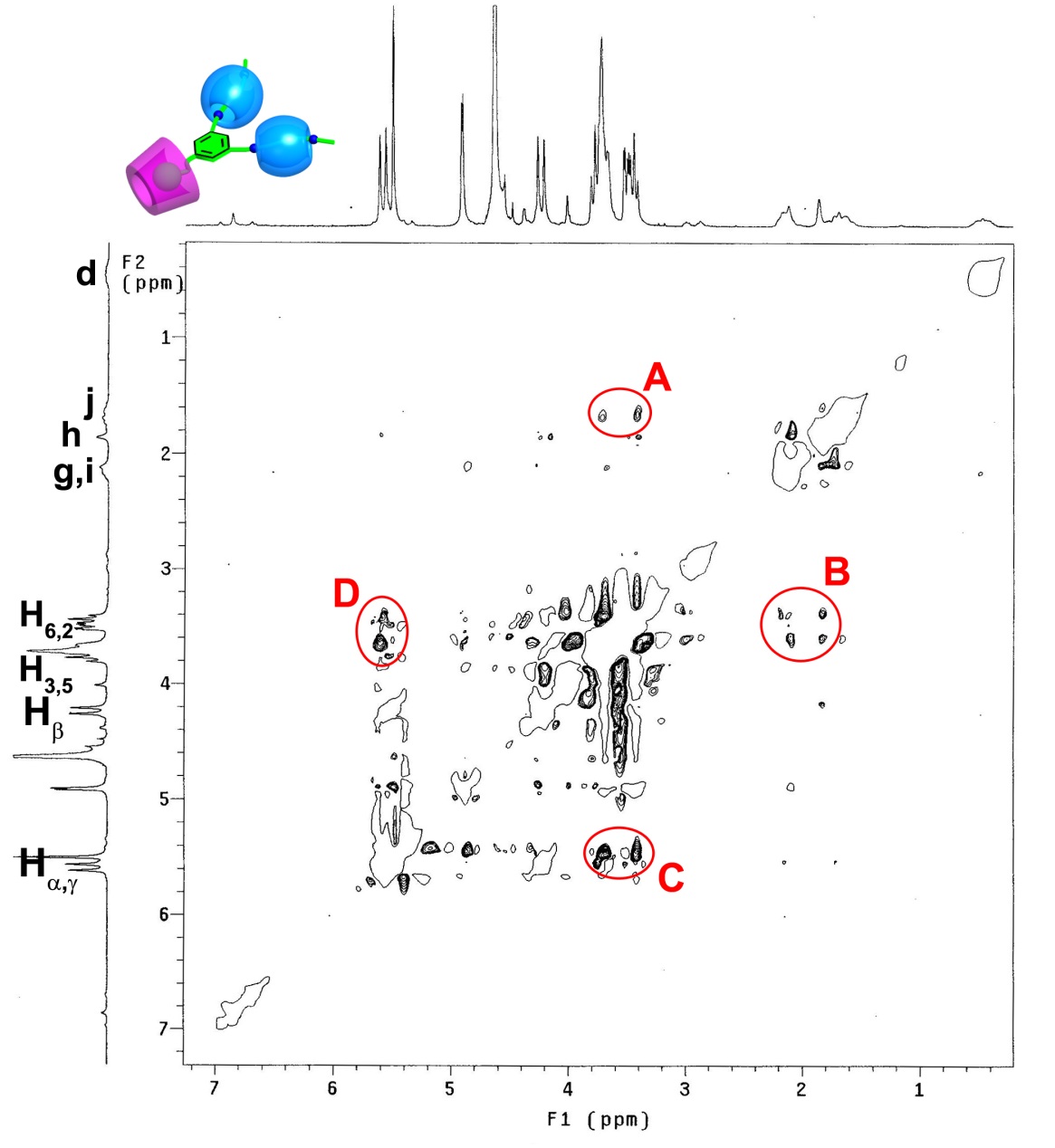


**Supplementary Figure S28.** NOESY spectrum of CB[6]‒ADA‒HACD conjugate with a mixing time of 0.200 s in D_2_O (400 MHz, [ADA] = 1.0 mM, [CB[6]] = 2.0 mM, and [*β*-CD] = 3.0 mM).


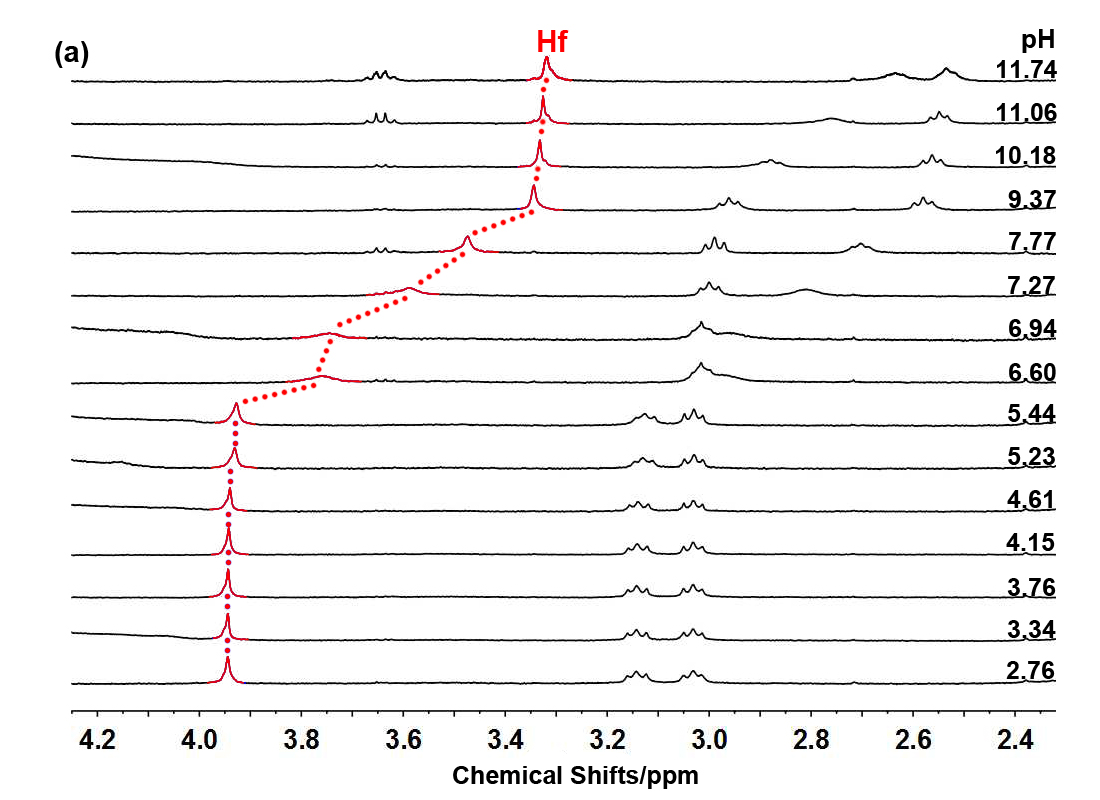


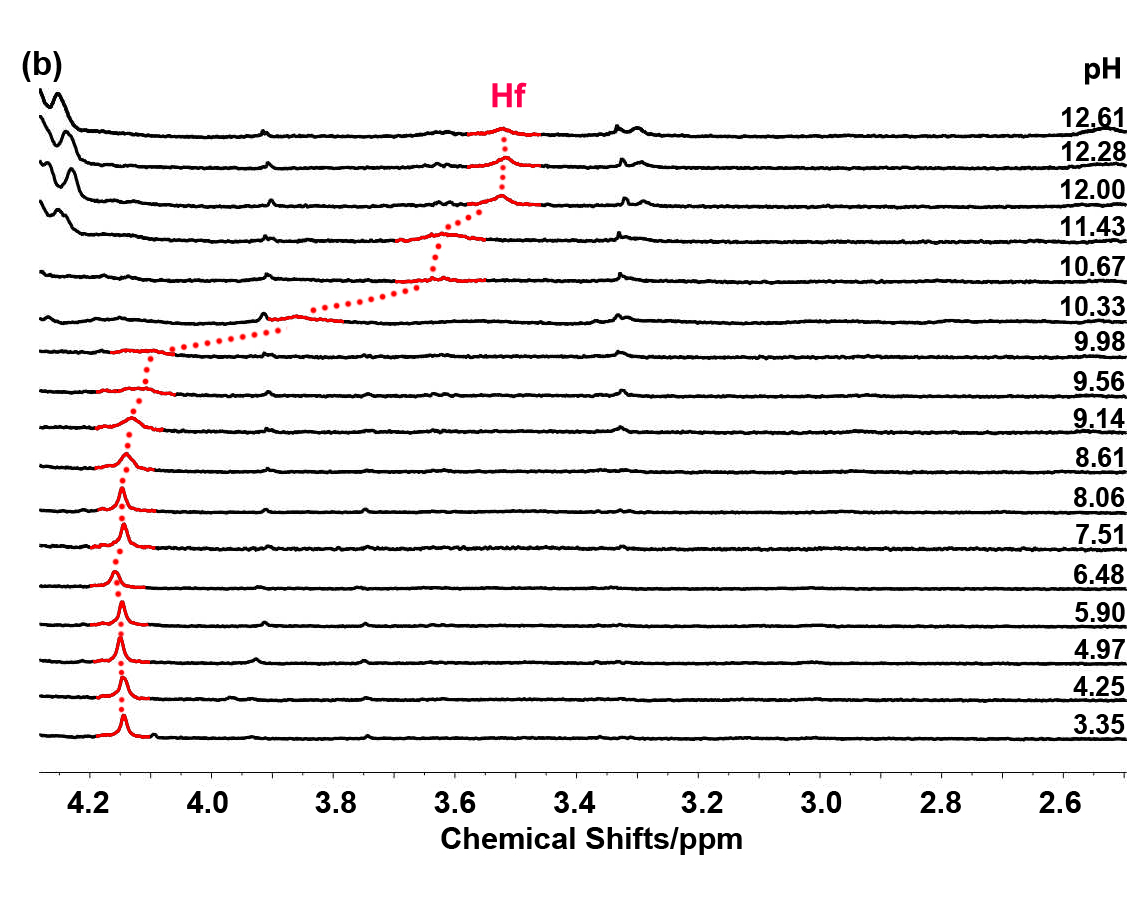


**Supplementary Figure S29.** ^1^H NMR spectral changes of H_f_ in (a) ADA and (b) ADA‒CB[6] complex upon pH variation at 25 °C (D_2_O, 400 MHz, [ADA] = 0.5 mM, and [CB[6]] = 1.0 mM).


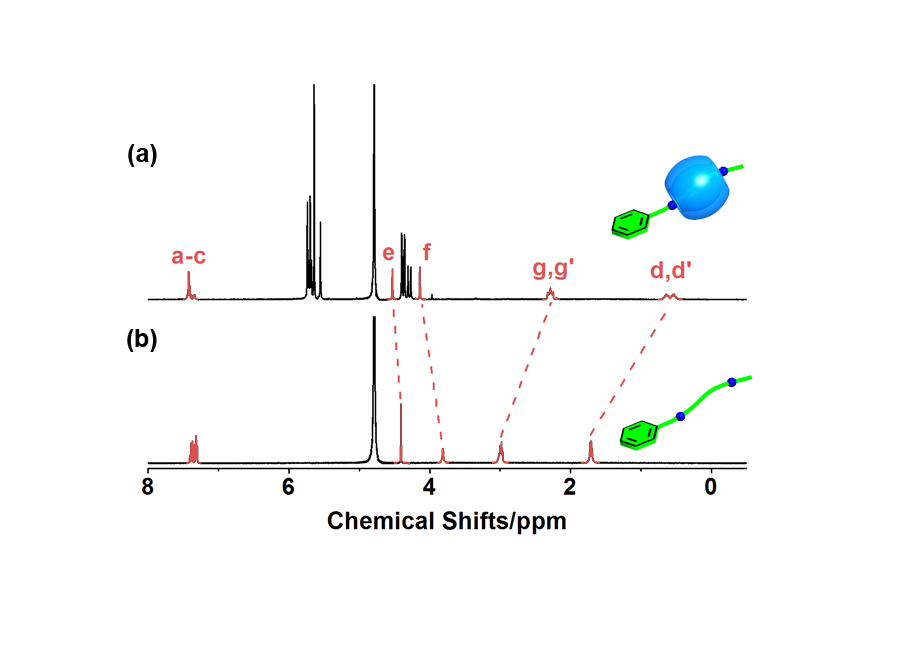


**Supplementary Figure S30.** ^1^H NMR spectra at 400 MHz of (a) CB[6]‒DAB complex and (b) ADA, in D_2_O at 25 °C ([DAB] = 2.0 mM and [CB[6]] = 2.0 mM).

**Supplementary Figure S31.** High-resolution ESI mass spectrum of CB[6]‒ADA complex in water. The peak at *m*/*z* 645.2487 corresponds to [ADA ‒ 4Br^‒^ + 2CB[6]]^4+^ (the expected mass is 645.2563).


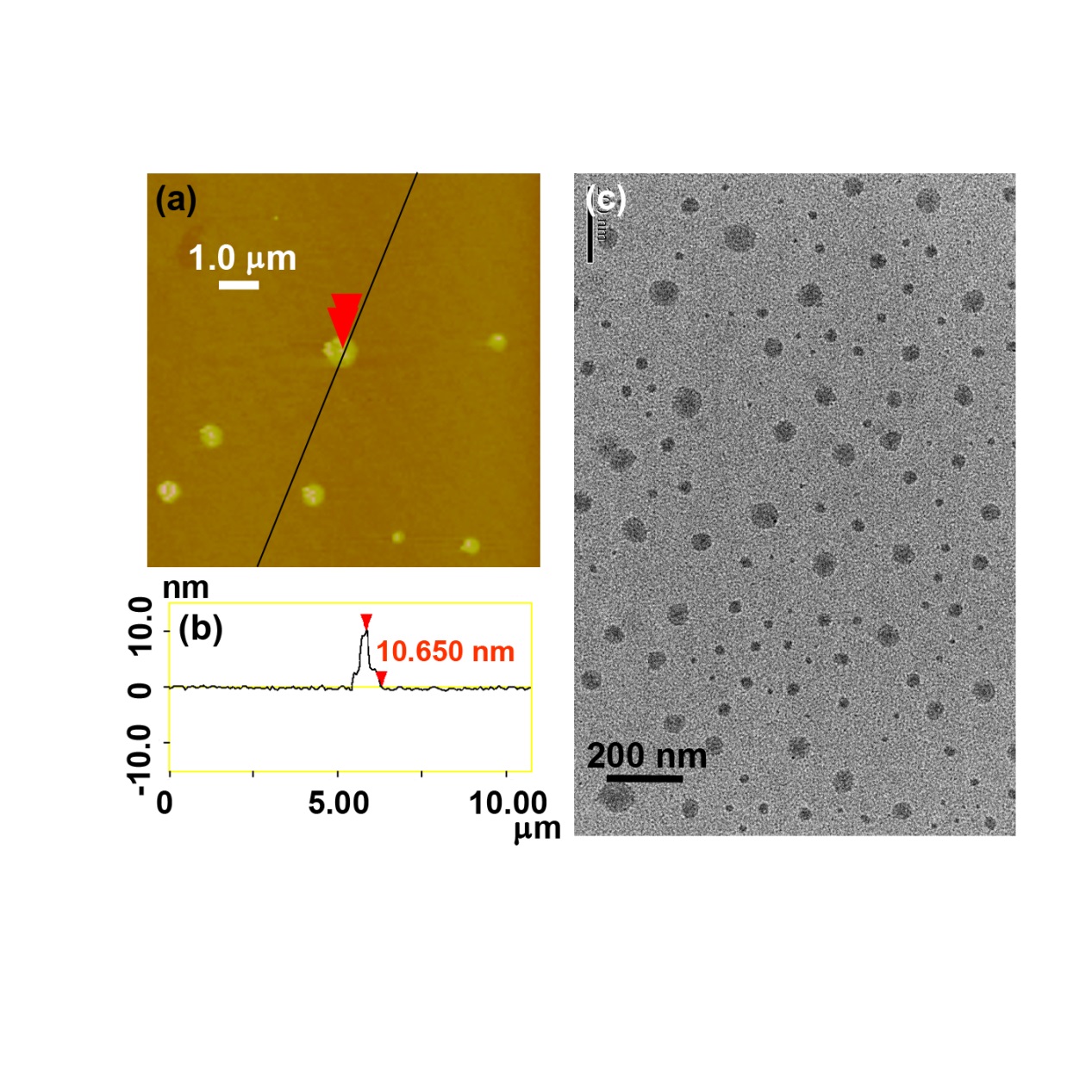


**Supplementary Figure S32.** (a) AFM image, (b) cross-section analyses, and (c) TEM image of CB[6]‒ADA‒HACD triads. Please note that the width measured in AFM image appeared larger than its real size due to the broadening effect of AFM tip[[3]](#endnote-4),[[4]](#endnote-5), whereas the nanoparticle’s width value can be accurately measured in TEM image.


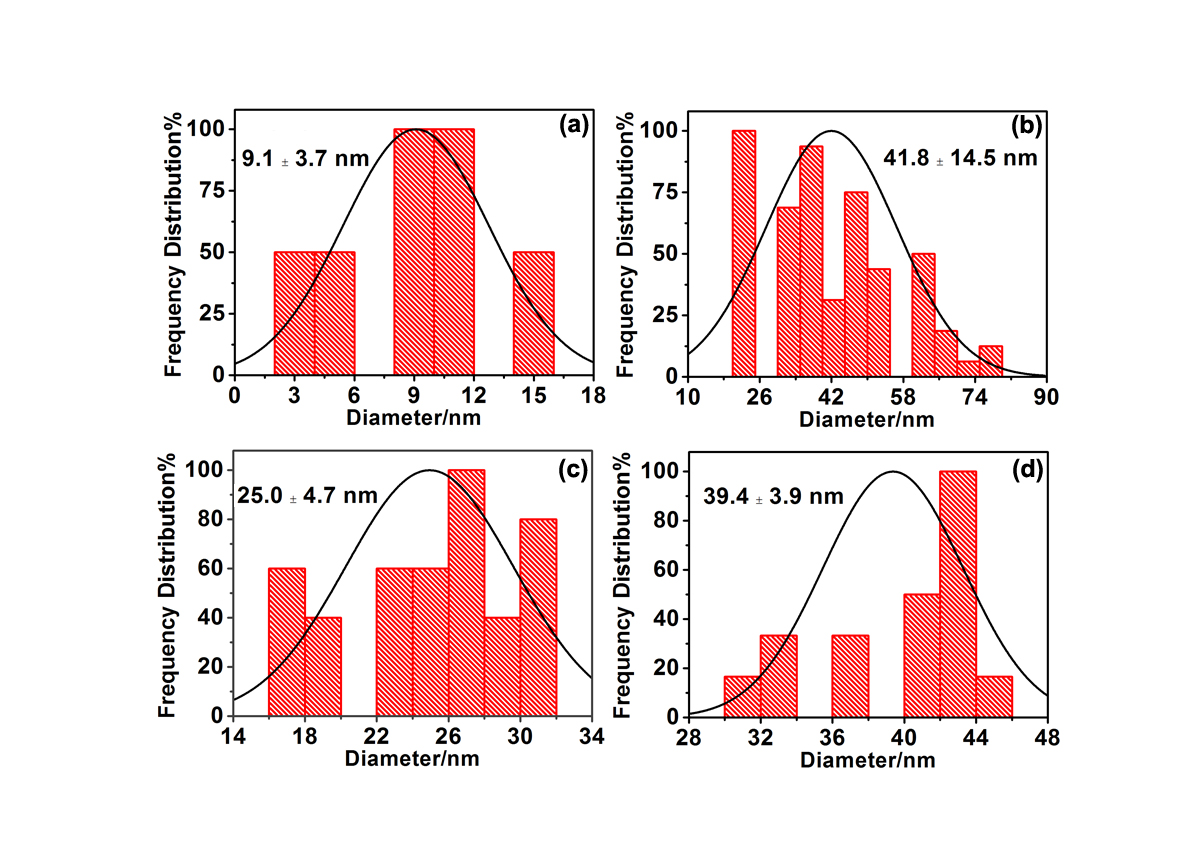


**Supplementary Figure S33.** Size distributions of (a, b) CB[6]‒ADA‒HACD triad and (c, d) its siRNA-bound polyplex, evaluated by (a, c) AFM and (b, d) TEM image statistics. Please note that although TEM statistics showed a similar width value (41.8 nm vs. 39.4 nm), the height value of ternary supramolecular complex measured in AFM image was much lower than that of its corresponding siRNA polyplexes (9.1 nm vs. 25.0 nm), thus giving a loose nanoparticulate with high aspect ratio in CB[6]‒ADA‒HACD complex.


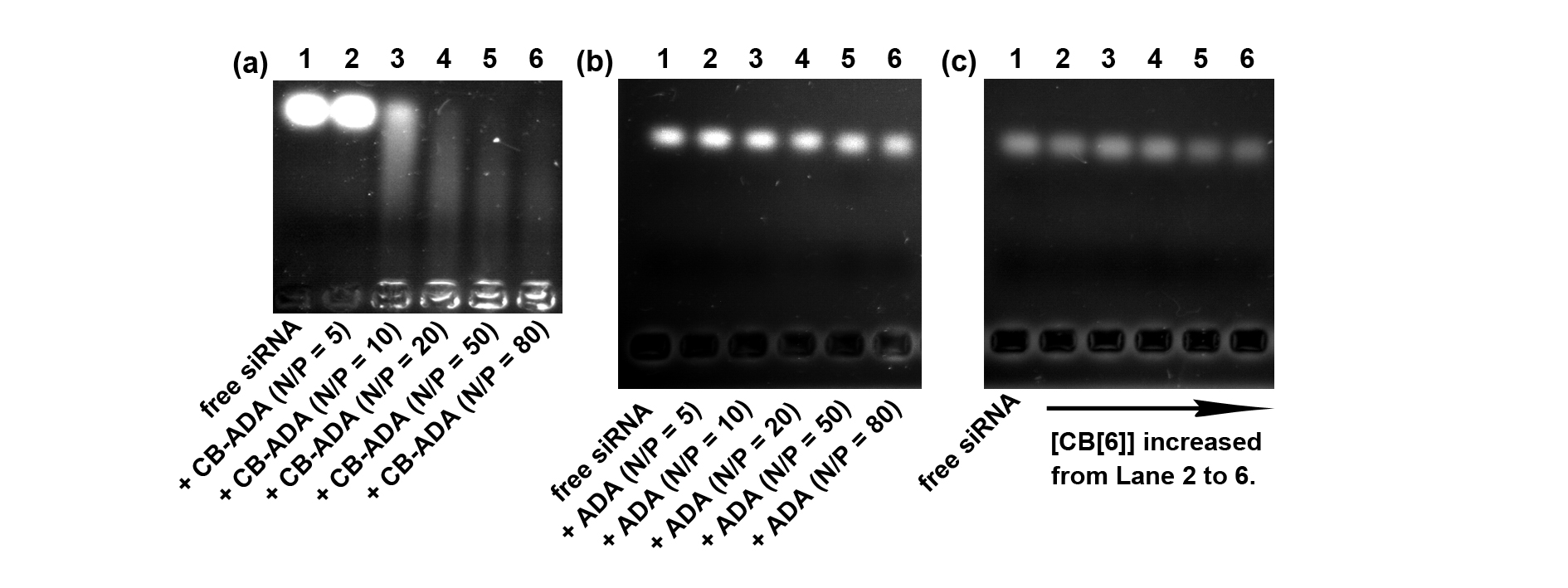


**Supplementary Figure S34.** Agarose gel electrophoresis assay of ADA, CB[6], and their complexes with negative control siRNA; the ADA:CB[6] ratio in (a) was fixed at 1:2. Compared to the ADA‒CB[6] complex in (a), the control experiments using the same concentrations of individual ADA and CB[6] were shown in (b) and (c), respectively.


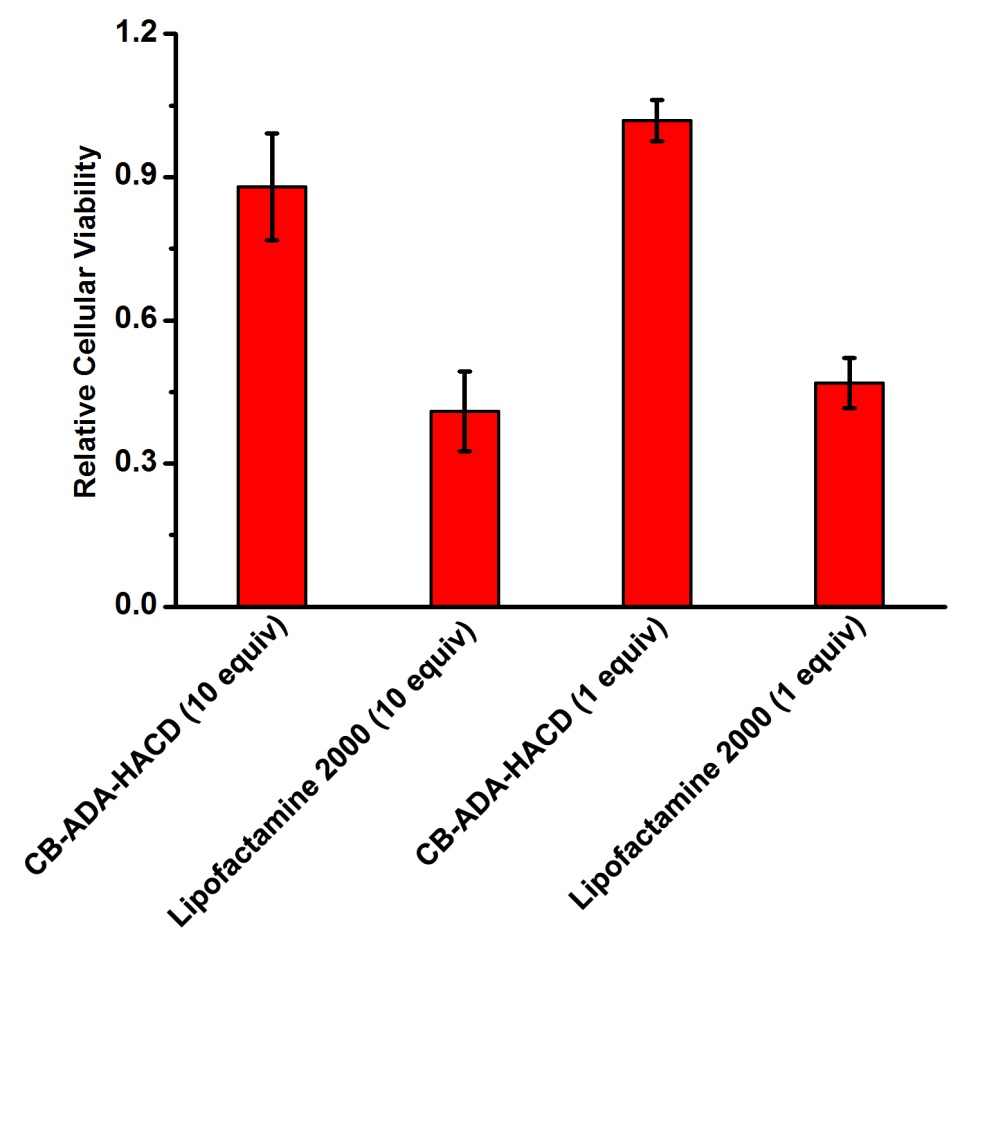


**Supplementary Figure S35.** Relative cellular viability of PC-3 cells after adding different concentrations of CB[6]‒ADA‒HACD assembly and Lipofectamine 2000. The differences that were statistically significant are indicated with asterisks (*p* < 0.05).


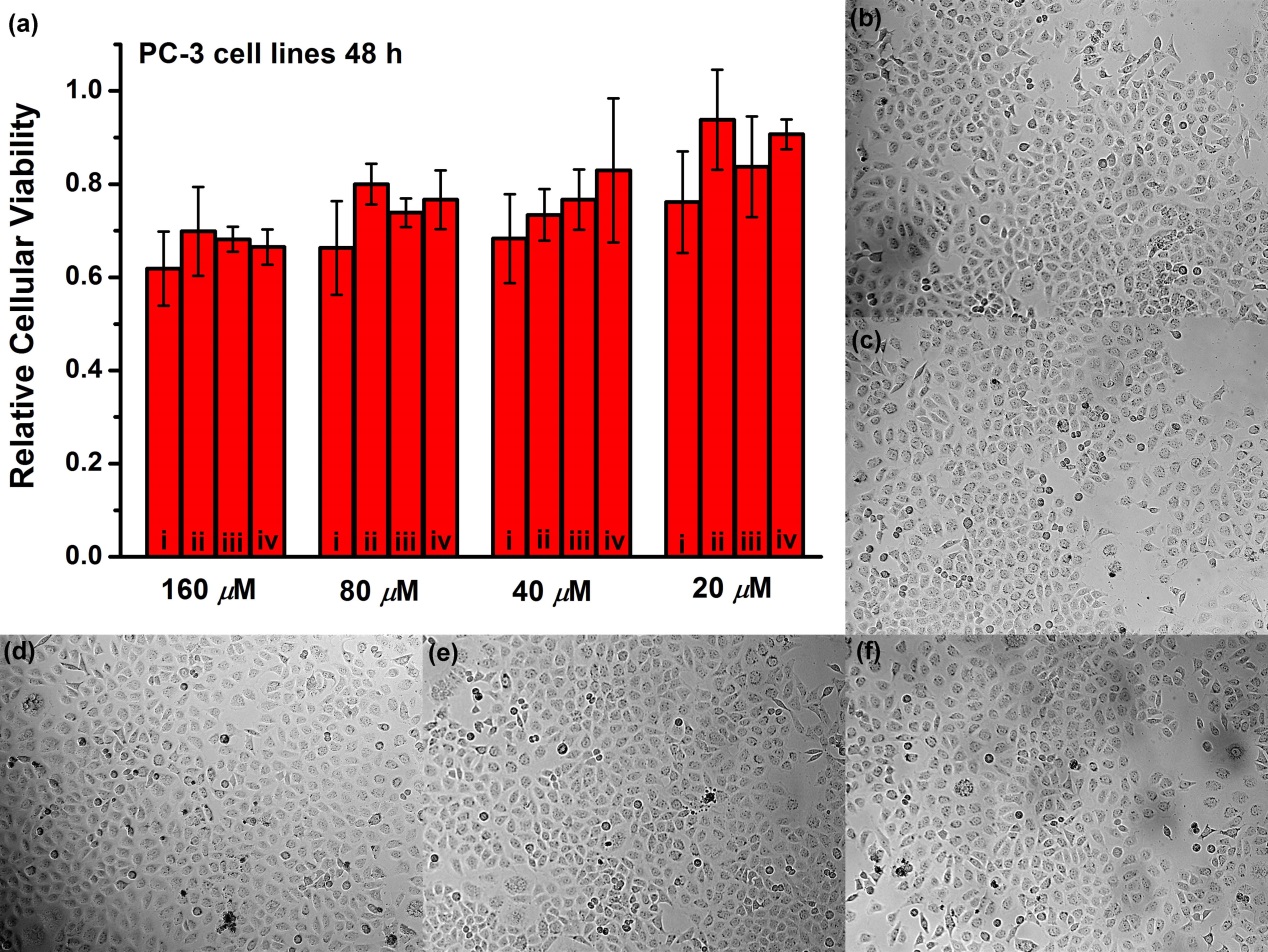


**Supplementary Figure S36.** (a) Relative cellular viability of PC-3 cells after addition of (i) ADA, (ii) ADA + CB[6], (iii) ADA + HACD, and (iv) CB[6] + ADA + HACD of different concentrations after 48 h. The differences that were statistically significant are indicated with asterisks (*p* < 0.05). Photographs of PC-3 cells upon addition of (c) ADA, (d) ADA + CB[6], (e) ADA + HACD, and (f) CB[6] + ADA + HACD, (b) blank control.


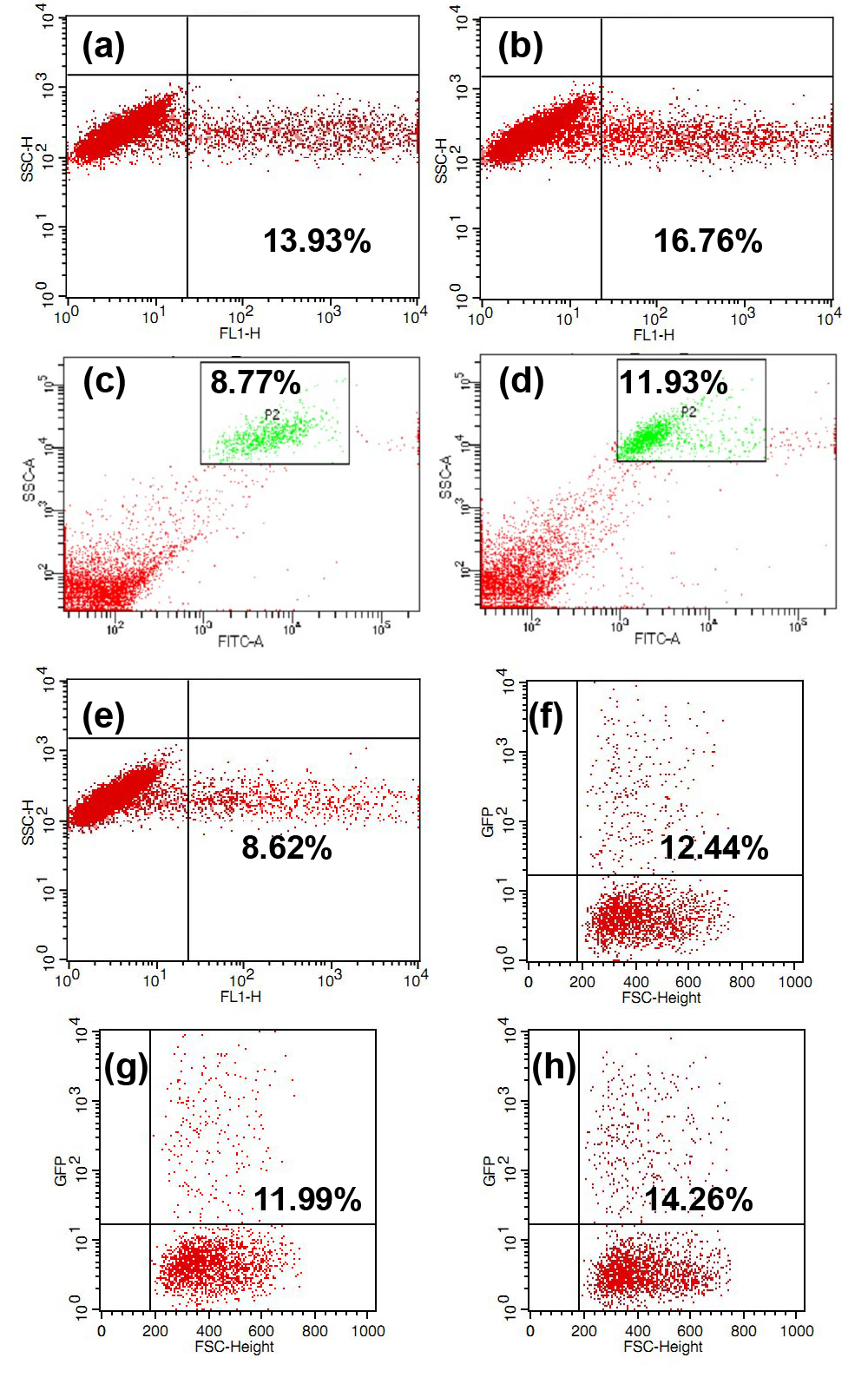


**Supplementary Figure S37.** Flow cytometric analysis of EGFP gene silencing effect in PC-3 cells. (a) negative control; and Lipofectamine 2000 + EGFP-pDNA followed by (b) Lipofectamine 2000 + EGFP-siRNA, (c) Lipofectamine RNAiMAX + EGFP-siRNA, (d) X-tremeGENE + EGFP-siRNA, (e) experimental group, (f) CB[6]‒ADA‒HACD + HA + EGFP-siRNA, (g) CB[6]‒ADA + EGFP-siRNA, and (h) HACD‒ADA + EGFP-siRNA, respectively.

**Supplementary References.**

1. Kim, S.-Y. *e**t al.* Toward high-generation rotaxane dendrimers that incoeporate a ring compoent on every branch: noncovalent synthesis of a dendritic [10]pseudorotaxane with 13 molecular components. *Chem. Asian. J.* **2**, 747–754 (2007). [↑](#endnote-ref-2)
2. Lee, J. W., Ko, Y. H., Park, S.-H., Yamaguchi, K. & Kim, K. Novel pseudorotaxane-terminated dendrimers: supramolecular modification of dendrimer periphery. *Angew. Chem. Int. Ed.* **40**, 746–749 (2001). [↑](#endnote-ref-3)
3. Kirby, A. R., Gunning, A. P. & Morris, V. J. Imaging polysaccharides by atomic force microscopy. *Biopolymers* **38**, 355–366 (1996). [↑](#endnote-ref-4)
4. Rivetti, C. & Codeluppi, S. Accurate length determination of DNA molecules visualized by atomic force microscopy: evidence for a partial B- to A-form transition on mica. *Ultramicroscopy* **87**, 55–66 (2001). [↑](#endnote-ref-5)
